# An Integrative Data Mining and Omics-Based Translational Model for the Identification and Validation of Oncogenic Biomarkers of Pancreatic Cancer

**DOI:** 10.3390/cancers11020155

**Published:** 2019-01-29

**Authors:** Nguyen Phuoc Long, Kyung Hee Jung, Nguyen Hoang Anh, Hong Hua Yan, Tran Diem Nghi, Seongoh Park, Sang Jun Yoon, Jung Eun Min, Hyung Min Kim, Joo Han Lim, Joon Mee Kim, Johan Lim, Sanghyuk Lee, Soon-Sun Hong, Sung Won Kwon

**Affiliations:** 1 College of Pharmacy, Seoul National University, Seoul 08826, Korea; phuoclong@snu.ac.kr (N.P.L.); 2018-23140@snu.ac.kr (N.H.A.); supercanboy@snu.ac.kr (S.J.Y.); mje0107@snu.ac.kr (J.E.M.); snuhmkim04@snu.ac.kr (H.M.K.); 2 Department of Biomedical Sciences, College of Medicine, Inha University, 3-ga, Sinheung-dong, Jung-gu, Incheon 400-712, Korea; inhafuture@gmail.com (K.H.J.); yanhonghua69@hotmail.com (H.H.Y.); 3 School of Medicine, Vietnam National University, Ho Chi Minh 70000, Vietnam; trandiemnghi@gmail.com; 4 Department of Statistics, Seoul National University, Seoul 08826, Korea; inmybrain@snu.ac.kr (S.P.); johanlim@snu.ac.kr (J.L.); 5 Department of Medicine, College of Medicine, Inha University, 3-ga, Sinheung-dong, Jung-gu, Incheon 400-712, Korea; limjh@inha.ac.kr (J.H.L.); jmkpath@inha.ac.kr (J.M.K.); 6 Division of Life and Pharmaceutical Sciences, Ewha Womans University, Seoul 120-750, Korea; sanghyuk@ewha.ac.kr

**Keywords:** pancreatic ductal adenocarcinoma, systems biology, meta-analysis, machine learning, next-generation sequencing, transcriptomics, diagnostic biomarker, prognostic biomarker

## Abstract

Substantial alterations at the multi-omics level of pancreatic cancer (PC) impede the possibility to diagnose and treat patients in early stages. Herein, we conducted an integrative omics-based translational analysis, utilizing next-generation sequencing, transcriptome meta-analysis, and immunohistochemistry, combined with statistical learning, to validate multiplex biomarker candidates for the diagnosis, prognosis, and management of PC. Experiment-based validation was conducted and supportive evidence for the essentiality of the candidates in PC were found at gene expression or protein level by practical biochemical methods. Remarkably, the random forests (RF) model exhibited an excellent diagnostic performance and *LAMC2*, *ANXA2*, *ADAM9*, and *APLP2* greatly influenced its decisions. An explanation approach for the RF model was successfully constructed. Moreover, protein expression of LAMC2, ANXA2, ADAM9, and APLP2 was found correlated and significantly higher in PC patients in independent cohorts. Survival analysis revealed that patients with high expression of *ADAM9* (Hazard ratio (HR)_OS_ = 2.2, *p*-value < 0.001), *ANXA2* (HR_OS_ = 2.1, *p*-value < 0.001), and *LAMC2* (HR_DFS_ = 1.8, *p*-value = 0.012) exhibited poorer survival rates. In conclusion, we successfully explore hidden biological insights from large-scale omics data and suggest that *LAMC2*, *ANXA2*, *ADAM9*, and *APLP2* are robust biomarkers for early diagnosis, prognosis, and management for PC.

## 1. Introduction

Pancreatic cancer (PC) is the seventh leading cause of cancer-related death worldwide, with less than seven percent of patients surviving after five years from the initial diagnosis [1]. Early diagnosis at a curable stage of PC is exceptionally challenging. Resection surgery may increase the 5-year survival rate to 20%, but only 10–20% of the patients are eligible for this procedure [2]. The symptoms of PC are usually not apparent due to its location deep inside the abdomen, and there are no current biomarkers with sufficient sensitivity and specificity for accurate diagnosis [3]. The cut-off level of carbohydrate antigen (CA) 19-9 for differentiating malignant and benign tumors is still controversial due to unconvincing diagnostic values. Additionally, the sensitivity of CA 19-9 drops dramatically in early and small tumors [4]. The lack of effective screening strategies and the paucity of powerful systematic therapies are also significant causes of PC fatality [5]. Collectively, novel methods for early detection and effective management of PC are urgently needed.

Only a small fraction of an enormous number of cancer biomarkers has been successfully implemented in the clinical practice. Cancer is characterized by tumor heterogeneity and a variety of molecular biosignatures; this calls for a more effective strategy of simultaneously combining a set of biomarkers [6]. A study has been reported on the improvement in diagnostic sensitivity of cancer using panels of combined biomarkers rather than individual molecules [7]. In PC, novel approaches applying methylated DNAs, circulating tumor DNAs, miRNAs, proteins, and metabolites have been developed and used with CA 19-9 in an attempt to enhance diagnostic and prognostic performance [8,9,10]. For instance, the sensitivity and specificity of the combined CA 19-9 and serum cytokines panel for differentiating PC from the normal group were 85.7% and 92.3%, respectively, and those of the combined CA19-9, ICAM-1, and OPG panel were 88% and 90%, respectively [11,12]. However, it is worth mentioning that several confounding factors may affect the establishment of a cancer biomarker panel. The samples for research on PC usually stem from patients with the advanced disease, as most patients are diagnosed at late stages, whereas biomarkers are expected to be for early, curable stages [13]. Additional clinical parameters, notably jaundice, are also worth considering when recruiting PC samples for biomarker studies [14]. 

Recent large-scale gene expression data studies have been conducted to shed light on molecular characterization and to provide a system-level view of human cancers, with a minor focus on PC [15,16]. Remarkably, a compendium including 2516 genes that showed changes in expression levels associated with PC was investigated, which accounted for nearly 13% of the known human coding genes [17]. However, the huge data load makes it impossible to translate the results into clinical applications. In this context, a promising and practical set derived from this database is needed. In the current study, we applied high-throughput next-generation sequencing (NGS)-based gene expression analysis, transcriptome meta-analysis, various data mining techniques with the previously published compendium and databases, and immunohistochemistry (IHC) on various cohorts for candidate validation to introduce multiplex signatures capable of differentiating PC tissue from the normal tissue. A powerful statistical learning technique and easy-to-comprehend explanation approach, using local interpretable model-agnostic explanations algorithm, were also utilized to enhance the diagnostic practice. The impact of the proposed biomarkers on patients’ survival was examined using The Cancer Genome Atlas (TCGA) cohort. Ultimately, an exceptional panel of biomarkers was introduced and initially validated. We also aimed to systematically assess the most promising therapeutic targets for PC. Finally, the associated biological and functionally altered signaling cascades of the introduced biosignatures in PC carcinogenesis have been discussed.

## 2. Results

### 2.1. Integrative Screening Approach Suggests 23 Novel Biomarker Candidates for the Diagnosis and Management of Pancreatic Cancer

**Figure 1:**
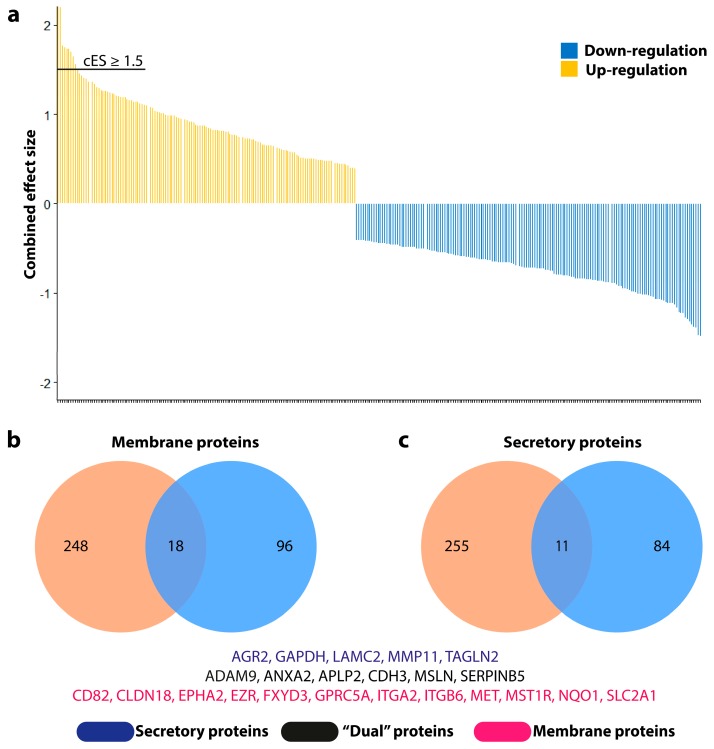
Screening process of innovative biomarker candidates for pancreatic cancer diagnosis and treatment. (**a**) Combined effect size distributions in transcriptome meta-analysis. (**b**) Selection of membrane protein-coding genes. (**c**) Selection of secretory protein-coding genes. The left circle of the Venn diagram (orange) represents the candidates from meta-analysis and the right circle of the Venn diagram (blue) represents the candidates curated from [17].

We conducted an effect size meta-analysis from two datasets to detect differentially expressed genes. Dataset GSE16515 included 36 cancerous samples and 16 adjacent normal tissues. Dataset GSE28735 contained 45 matching pairs of pancreatic cancer and adjacent non-tumor tissues. Table S1 lists the characteristics of all utilized microarray data. We identified 3915 significantly upregulated and 4007 significantly downregulated genes in the PC tissue compared to normal tissue. It is worthy to note that we targeted upregulated genes in an effort to identify oncogenic biomarkers for the diagnosis, prognosis, and treatment of PC. It turned out that less than 7% (266 out of 3915) of overexpressed genes demonstrated a combined effect size (cES) greater than or equal to 1.5, the selection criterion for potential candidates (Figure 1a). In parallel, we utilized an exhaustive compendium of PC biomarkers collected by Harsha et al. as the filtering resource [17]. The genes coding for secretory proteins and membrane proteins overexpressed in PC, precursor lesions, the stroma associated with PC, and other subtypes of PC, but not in pancreatitis, were selected. Of note, we tried to limit our study to genes that are only overexpressed in PC but not in chronic pancreatitis, which are also those having potential to distinguish PC from chronic pancreatitis, since it may be difficult to distinguish chronic pancreatitis from PC in some cases, as there have been no excellent biomarkers to distinguish these two conditions [17,18]. Given these criteria, 95 secretory and 114 membrane protein-coding genes from the compendium fulfilled the criteria. Based on the Venn diagram analysis of the candidates from the meta-analysis and the compendium, five secretory (*AGR2*, *GAPDH*, *LAMC2*, *MMP11*, and *TAGLN2*), 12 membrane (*CD82*, *CLDN18*, *EPHA2*, *EZR*, *FXYD3*, *GPRC5A*, *ITGA2*, *ITGB6*, *MET*, *MST1R*, *NQO1*, and *SLC2A1*), and six “dual” (*ADAM9*, *ANXA2*, *APLP2*, *CDH3*, *MSLN*, and *SERPINB5*) protein-coding genes were selected for further analysis (Figure 1b,c).

### 2.2. Literature- and Experiment-Based Validation of the 23 Selected Candidates

Published papers and data submitted to public repositories were extensively mined for literature-based validation. The overexpression of the 23 candidates in pancreatic ductal adenocarcinoma (PDAC) was experimentally identified and validated by multiple techniques, such as microarray, quantitative proteomics, and antibody-based methods. According to the results, each gene was overexpressed with a change greater than two-fold at least once in PC or PC cell lines at the mRNA or protein level. For instance, *ANXA2* was preferably reported to be overexpressed in PDAC tissue in the mRNA analysis using DNA Microarray and in the protein analyses using IHC, isotope-coded affinity tag, western blot, mass spectrometry, two-dimensional electrophoresis, and label-free quantitation mass spectrometry. 

We further conducted an NGS gene expression analysis on a cohort containing six PDAC samples and six controls for experiment-based validation. Despite the heterogeneity and other confounding factors, the NGS result was promising, showing 17 out of 23 consistently overexpressed genes with a cut-off of two-fold change (log FC = 1.00), and 14 out of 23 genes using log FC = 1.50. Remarkably, *SERPINB5* was more than 250 times overexpressed in the PC tissue compared to normal tissue (log FC = 8.35). *LAMC2*, *CDH3*, and *ANXA2* exhibited the altered expression difference with log FC of 3.91, 2.72, and 1.16, respectively. Collectively, the experimental and in silico analyses largely supported our 23 selected candidates as reliable biomarkers for differentiating PC tissue from normal tissue. 

Genetic alterations occur commonly in patients with curable pancreatic pre-malignant lesions, making the biomarker discovery at early stages indispensable [19]. The altered expression of the 23 genes in pancreatic intraductal papillary-mucinous neoplasm (IPMN) and intraductal papillary-mucinous carcinoma (IPMC) was also explored. Among the selected candidates, *AGR2*, *LAMC2*, *TAGLN2*, *MSLN*, *SERPINB5*, *CLDN18*, *EZR*, *FXYD3*, *GPRC5A*, *ITGA2*, *MST1R*, *NQO1*, and *SLC2A1* were upregulated in pancreatic neoplasms compared to normal tissue, while *CDH3*, *EPHA2*, *GPRC5A*, and *SLC2A1* were upregulated in PC compared to pancreatic neoplasms, providing clues that they may be involved in PC initiation and development. 

The list of techniques for gene expression, identification, and validation, cES, and gene expression in premalignant pancreatic lesions are summarized in Table 1. 

### 2.3. ADAM9, ANXA2, ITGA2, MET, and LAMC2 Exhibit Substantial Impact on the Survival of PC Patients

**Figure 2:**
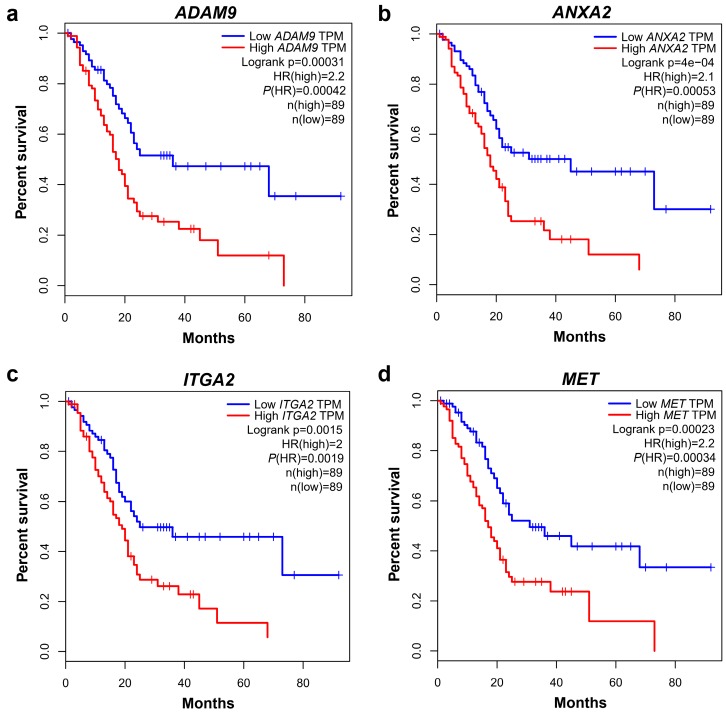
Kaplan–Meier (KM) plots of the overall survival of four promising prognostic candidates. (**a**) KM plot of *ADAM9*. (**b**) KM plot of *ANXA2*. (**c**) KM plot of *ITGA2*. (**d**) KM plot of *MET*.

The Kaplan-Meier (KM) survival analysis and the multivariate Cox regression analysis were conducted based on the available data from TCGA pancreatic cancer cohort. The KM survival analysis revealed that PC patients with high expression levels of 17 of the candidate genes exhibited a poorer overall survival (OS) or disease-free survival (DFS). Particularly, patients overexpressing *ADAM9*, *ANXA2*, *ITGA2*, or *MET*, had both poorer OS and DFS rates than those with low gene expression. Figure 2 shows the KM plots of the OS for *ADAM9*, *ANXA2*, *ITGA2*, and *MET* expression. Of note, the HR was greater than 2 for both analyses, except for *ITGA2* that had an HR of 1.9 for the DFS analysis. *LAMC2* exhibited a significantly poor DFS (HR = 1.8, *p*-value = 0.012). A multivariate Cox regression analysis of the TCGA cohort was adopted to evaluate the impact of the 23 genes in patient survival. The analysis revealed that *LAMC2*, *ADAM9*, *ANXA2*, *CDH3*, *SERPINB5*, *EPHA2*, *GPRC5A*, *ITGA2*, *ITGB6*, and *MET* were likely associated with the worse outcome of PC with FDR < 0.05. The detailed information regarding Cox regression survival analysis of these genes is described in Table 2 and Figure S1.

### 2.4. Supervised Machine Learning Classification Demonstrates that ADAM9, ANXA2, LAMC2, and APLP2 Are Important in Differentiating PC Tissue from Normal Tissue

**Figure 3:**
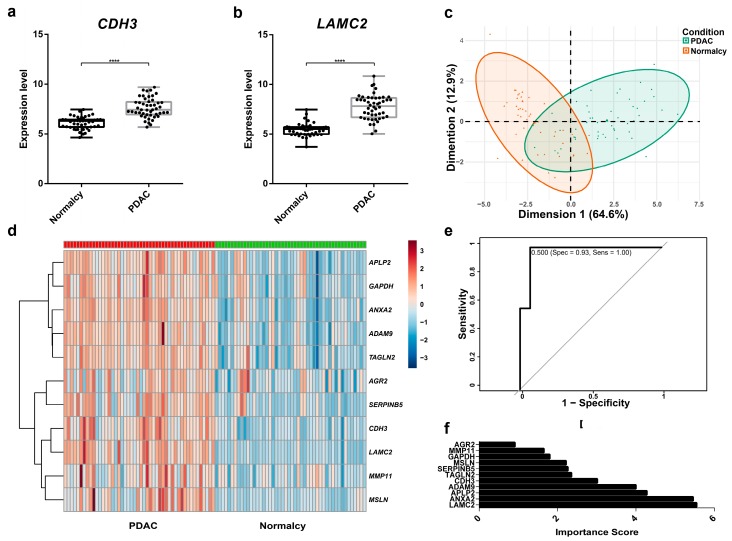
Data exploration and diagnostic performance of 11 biomarker candidates in the Random Forests model. (**a**) The gene expression of *CDH3* is higher in PDAC than in normal controls (*n* = 96), ****: *p* < 0.0001. (**b**) The gene expression of *LAMC2* is higher in PDAC than in normal controls (*n* = 96). (**c**) Principal component analysis of PDAC versus normal controls. (**d**) Heatmap analysis of 11 biomarker candidates. (**e**) ROC curve of the random forests model in the test set (*n* = 28). (**f**) The importance scores of 11 biomarker candidates in the random forests model.

The role of 11 secretory protein-coding genes for differentiating PC tissue from normal tissue was further tested. We visualized the expression profiling of the 11 genes by means of boxplots. For instance, the expression levels of two promising candidates, *CDH3* and *LAMC2*, were significantly higher in PDAC compared to normal tissue (Figure 3a,b). Boxplots of the remaining potential genes can be found in Figure S2. As illustrated in Figure 3c, two classes of PC and normal tissues showed a relatively separated tendency in the principal component analysis. However, there was still an overlapped region between them, making grouping of those cases more challenging. A heatmap analysis further provided a general overview of relative differences in gene expression between PC and normal tissue (Figure 3d). As expected, in general, the 11 candidates exhibited a higher level of expression in PC tissues than in normal tissue. The dataset was then randomly divided into the training set (70%) and test set (30%) for supervised RF model calibration and validation. RF classification was conducted to build the prediction model based on 11 potential genes. The number of randomly chosen variables at each split ranged from one to 11, and the data were cross-validated tenfold to find the best tuning parameter, which was repeated five times. The optimal RF model (mtry of two) in our trial revealed an excellent performance with an area under the ROC curve of 0.96 in the training set. The model validated in the test set also exhibited promising results with only one misclassified case, reporting accuracy of 0.96, sensitivity of 1.00, and specificity of 0.93 (Figure 3e). Of note, *LAMC2*, *ANXA2*, *APLP2*, and *ADAM9* were the most important variables of the model, although other genes facilitated the decision (Figure 3f). 

Local Interpretable Model-Agnostic Explanations (LIME) algorithm was applied to explain the “decision rules” of our black-box model. The first case was classified into the normal group, with a probability of 0.96 based on the gene expression rules: *ANXA2* ≤ 9.77, *APLP2* ≤ 8.31, *ADAM9* ≤ 6.74, 5.31 < *LAMC2* ≤ 6.07, and 3.61 < *SERPINB5* ≤ 4.46. Another case was certainly predicted as PC based on the rules: *LAMC2* > 7.82, *ANXA2* > 10.62, *ADAM9* > 8.09, *MMP11* > 6.58, and *AGR2* > 9.68, reaching the probability of 1.00. We deduced from the typical cases that a higher expression level of *ANXA2* (cut-off threshold = 9.77), *ADAM9* (cut-off threshold = 6.74), *LAMC2* (cut-off threshold = 6.07), *AGR2* (cut-off threshold = 9.68), *MMP11* (cut-off threshold = 6.58), *APLP2* (cut-off threshold = 8.31), and *SERPINB5* (cut-off threshold = 4.46) was an indication of PC in this investigation. The visualization of the above explanations of the four representative cases is given in Figure S3. 

### 2.5. Immunohistochemistry Analysis Reveals High Protein Expression Levels of LAMC2, ADAM9, ANXA2, and APLP2 in Pancreatic Cancer

**Figure 4:**
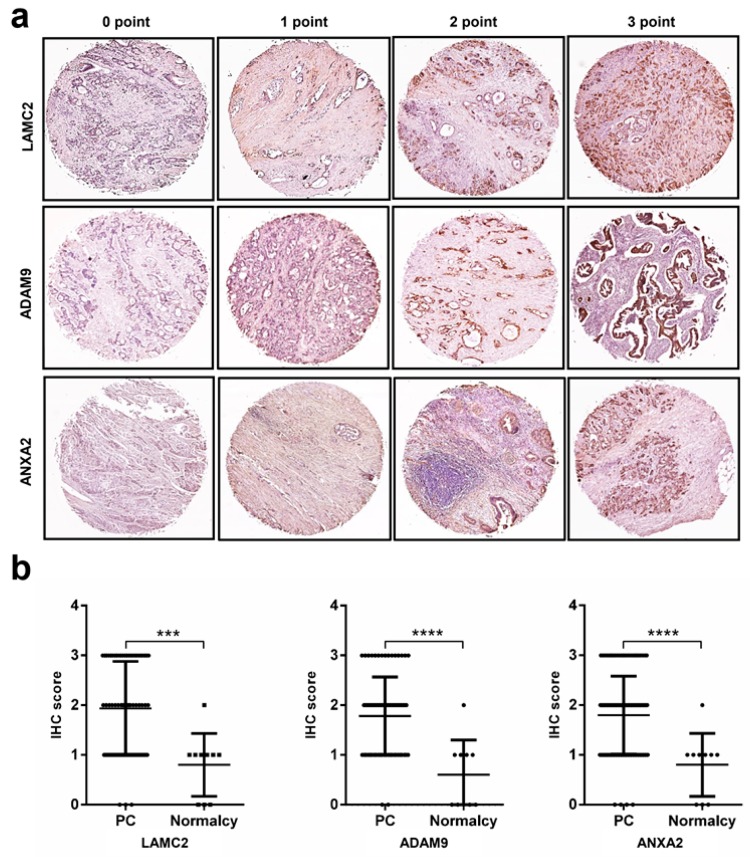

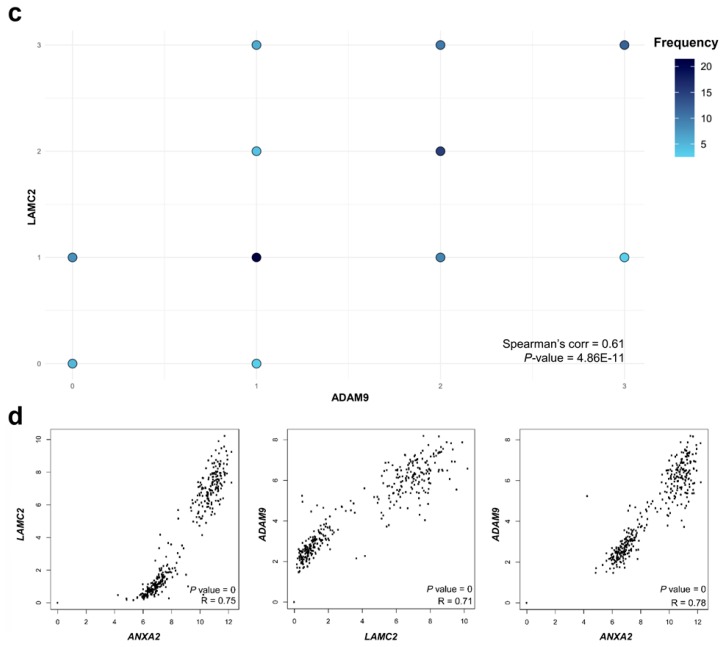
Immunohistochemical analysis for determination of *LAMC2*, *ADAM9*, and *ANXA2* (*Annexin A2*) gene expression. (**a**) Scoring system for the three proteins in pancreatic tissues. (**b**) IHC scores of *LAMC2*, *ADAM9*, and *ANXA2* in pancreatic tumors and normal controls (*n* = 86). ***: *p* < 0.001; ****: *p* < 0.0001. (**c**) Pairwise correlation of *LAMC2*, *ADAM9*, and *ANXA2* expression. (**d**) Pairwise correlation of *LAMC2*, *ADAM9*, and *ANXA9* at gene expression level (log_2_ of transcripts per million) in TGCA PC, TCGA normal pancreas, and Genotype-Tissue Expression Project (GTEx) derived normal pancreas.

We assessed the expression of *LAMC2*, *ADAM9*, and *ANXA2* at the protein level using tissue microarray samples of 86 patients and normal controls. They were chosen due to their roles in the diagnostic model, prognostic impact, and profound validated evidence. Typical immunostaining is shown in Figure 4a, and the cohort characteristics are given in Table S2. Individual proteins showed moderate to strong immunostaining in most PC tissues, and weak or negative immunostaining in normal tissues; the differences are statistically significant (Figure 4b, *p*-value < 0.001). *LAMC2*, *ADAM9*, and *ANXA2* exhibited moderate correlations with their protein levels according to Spearman’s pairwise correlation (Figure 4c, Figures S4 and S5). The protein expression of LAMC2, ADAM9, and ANXA2 between PC and adjacent tissues (eight paired samples) presented similar results, although only ADAM9 showed statistical significance (Figure S5, *p*-value = 0.031). The protein expression of APLP2 was also investigated in a cohort comprising 64 PC and 64 adjacent normal tissues (Table S3). As shown in Figure S6, the expression of APLP2 was significantly higher in PC tissues (*p*-value = 0.0139), making them valuable for further investigation. The Pearson’s correlation analysis of gene expression between *LAMC2*–*ADAM9* (*R* = 0.71, *p*-value < 0.001), *ANXA2*–*LAMC2* (*R* = 0.75, *p*-value < 0.001), and *ANXA2*–*ADAM9* (*R* = 0.78, *p*-value < 0.001) revealed strong correlations among them (Figure 4d). 

### 2.6. Functional Analysis and Public Data Mining to Create a Comprehensive Picture of the Biological Processes in Pancreatic Cancer

Cancer Hallmarks Analytics Tool (CHAT) was employed to clarify the involvement of our candidates in the biological processes leading to PC according to the current ten hallmarks of cancer. Surprisingly, 18 of the 23 genes were implicated in at least five cancer hallmarks, suggesting that they may be highly associated with PC carcinogenesis and progression. Finally, a pathway enrichment analysis was performed based on the 397 upregulated genes with a cES greater than 1.5, derived from the meta-analysis of five datasets, to provide solid evidence on the PC related biological processes. As a result, various KEGG-annotated pathways were enriched in the PC tissue compared to normal tissue, including prominent cancer-related pathways, such as cell cycle and intrinsic molecular processes of PC and others, such as prostate cancer and chronic myeloid leukemia. The results indicate both unique and overlapping features of PC and other cancers, which would be helpful for further studies. In the protein–protein interaction network of 397 upregulated genes, strong connections among the nodes were observed (score > 0.900). There were two major clusters formed by pathways in cancer and cell cycles in the network (Figure S7). Pathway enrichment, protein–protein networks with known and predicted interactions between the nodes, and CHAT analysis can be found in Tables S4 and S5, and Figure S7.

### 2.7. Drug–Gene Interaction and miRNA–Gene for Further Development of Novel Therapeutics

Increasing evidence suggests that aberrantly expressed miRNAs are attractive targets for preventing PC initiation, progression, metastasis, and chemoresistance. Given their enormous potential as therapeutic tools for cancer management, we checked whether the transcripts of our 18 membrane protein-coding genes were the targets of miRNAs based on experimentally validated evidence. It turned out that nine candidates exhibited a strong connection with miRNAs (*ADAM9*, *ANXA2*, *MSLN*, *SERPINB5*, *EZR*, *GPRC5A*, *ITGA2*, *MET*, and *SLC2A1*). The Pearson’s correlation coefficient between each candidate and the miRNA was also recorded. Finally, we input our genes in curated resources combined by the drug-gene interaction database (DGIdb) to examine if they coded for a drug-metabolizing enzyme or a drug transporter (so-called druggable genes), and then determined possible drug-gene interactions. As an example of the results, *ADAM9* was positively correlated with hsa-miR-126-3p (the correlation coefficient, 0.315; *p*-value < 0.05) with strong evidence from the reporter assay, western blot, and qPCR. According to DrugBank, the drug Ilomastat was detected to inhibit the cell-surface protein ADAM9. The detailed information regarding miRNA–gene interaction and drug–gene interaction is presented in Table 3. 

## 3. Discussion

PC is a complicated and highly heterogeneous disease, yet it takes approximately two decades for a primary pancreatic tumor to develop from initiation to cancer death, opening a large window for an effective screening and early detection program [20]. In that context, a plethora of biomarkers have been studied, but most of them have failed to be translated into clinical practice. Robust biomarkers, even identified by advanced, reliable technologies, have not exhibited a better diagnostic and prognostic ability than conventional biomarkers in large-scale clinical trials [21]. In recent years, advances in computational techniques have facilitated and enhanced cancer omics-based research [22]. The current study utilizes an unbiased data-driven approach together with the integration of omics data, machine learning, and conventional immunohistochemistry approaches to suggest and validate potential biomarkers for PC diagnostics and management. Our approach covers a broad spectrum of cancer biology, of which *LAMC2*, *ANXA2*, *ADAM9*, and *APLP2* are the most important features of the tested diagnostic model, and their implementation in clinical settings could be further examined. The roles of LAMC2, ANXA2, ADAM9, and APLP2 in cancer biology have been documented [23,24,25,26,27]. However, our study has been a pioneer in suggesting the combination of *LAMC2*, *ANXA2*, *ADAM9*, and *APLP2* and statistical learning algorithms to properly detect PDAC in early stages. Of note, the expression at the gene and protein level of *LAMC2*, *ANXA2*, and *ADAM9* are positively correlated to PC. PC patients overexpressing *LAMC2* potentially have a higher chance of getting distant metastasis and poorer prognosis [28]. The elevation of LAMC2 in serum was also considered an indicator for differentiating healthy people from an early stage of PDAC with outstanding performance [29]. Similarly, the high expression of stromal ANXA2 was significantly correlated with short disease-free survival and overall survival, while the high expression of ADAM9 was associated with poor tumor differentiation and short overall survival in PDAC patients [26]. We also screened for the presence of somatic mutations of our candidates using the most comprehensive global database for the annotation of somatic variants in human cancer, the Catalogue of Somatic Mutations in Cancer (COSMIC) [30]. COSMIC has systematically curated published cancer mutation data and provides in-depth knowledge on cancer genes, including their involvement in cancer genesis and mutation mechanisms. It turned out that our candidates were not frequently mutated genes. Further studies integrating the suggested biosignature and conventional biomarkers, such as CA 19-9, are needed to validate the performance of the panel. Nevertheless, our investigation demonstrates that with the power of machine learning and omics-based big data, scientists could gather patient tumor data and clinical parameters together with biomarker screening using the combined panel to explore the pattern of alterations in multiple PDAC patient tumors, thereby increasing the diagnostic and prognostic accuracy.

Out of the 23 genes in our panel, 18 were involved in at least five cancer hallmarks, indicating the relation of these genes and associated products with cancer initiation and progression. However, with respect to panel, the roles of immune destruction, cellular energetics, and replicative immortality in PC have not been studied frequently compared with the other hallmarks. More studies regarding these aspects are needed to fully explore the potential of our multiplex panels in PC diagnosis and treatment. In recent years, extensive efforts have been made to shed light on the association between immune destructions, cancer, and associated regulators [31,32]. The immune hallmark can be summarized as the ability to survive in the chronically infiltrated microenvironment, to escape the immune recognition, and to suppress the immune response [33]. Despite their great impact on cancer treatment and prevention, these processes may also give some clues for developing new screening and diagnostic approaches. Of note, the deregulating cellular energetics or cancer metabolism has been recently recognized as a cancer hallmark [34]. Investigations in the past 20 years and the successful implementation of metabolic tumor imaging into clinical practice have once again emphasized that altered metabolic processes are critical for tumor survival and proliferation [35]. Among them, the alterations of glycolysis, amino acids, and lipid metabolism are the most important changes of cancer cellular energetics [36]. Multiple metabolic enzymes and pathways could be investigated as attractive targets not only for anticancer therapeutics but also for developing novel biomarkers [37]. 

It was expected that miRNA would diminish drug resistance of tumors through various regulatory mechanisms [38,39]. In our investigation, *ADAM9* and *ANXA2* were attractive candidates for miRNA-based anti-PC therapies. They exhibited strong interactions with miR-126, miR-33a, and miR-125a (*ADAM9*); and miR-155 and miR-206 (*ANXA2*). In PC, miR-126 regulated *ADAM9* by targeting the epithelial-mesenchymal transition, the initial step of the metastatic cascade [40]. *ANXA2* and *KRAS* were also direct downstream targets of miR-206 that modulated cancer cell growth, metastasis, and lymphangiogenesis in PDAC [41]. In addition to miRNAs, various drug molecules are worthy of more attention. Lately, in an effort to implement evidence-based personalized treatment recommendations into clinical settings, some software and databases have been developed to assist the decision of PC treatment. A recent evidence mining software analyzing next-generation sequencing data suggests pharmacogenomic biomarkers and has potential to predict PC treatment efficacy and toxicity [42]. Further studies are necessary to fully explore the potential of *ADAM9* and *ANXA2*, with high level of evidence of clinical validity, among other candidates, as drug candidates in PC management with regard to their associated miRNAs.

The current study set out to introduce an integrative data mining model for translational research to facilitate the discovery and validation of new biomarkers. In particular, a comprehensive literature and experimental data mining approach to examine potential roles of overexpressed genes as biomarkers for diagnosis and prognosis of pancreatic cancer were conducted. Thus, as a limitation, down-regulated genes were neglected in all of our analyses, which might result in the missing of valuable candidates for further validation. In addition, a follow-up study integrating four proposed candidates and statistical learning is needed to examine their actual clinical validity.

## 4. Materials and Methods

### 4.1. Ethical Approval and Consent of Patients

The Institutional Review Board (IRB) of Seoul National University reviewed and exempted our protocol for a full ethical approval application when using human gene expression data from the public database without personal information, survival data, and commercial tissue micro-array (SNU 16-11-014 and SNU 17-05-043). The IRB of Inha University approved the protocol of the NGS gene expression analysis (INHAUH 2016-08-008-001). Every patient signed an informed consent before any procedures were conducted. The experiments followed the Ethical Principles of the World Medical Association Declaration of Helsinki.

### 4.2. Antibodies

ADAM9 antibody was purchased from Invitrogen (PA5-25959, Waltham, MA, USA), Annexin A2 (ANXA2) antibody was purchased from Cell Signaling (#8235, Beverly, MA, USA), and LAMC2 and APLP2 antibodies were purchased from Sigma (AMAb91098 and HPA039319, St. Louis, MO, USA). 

### 4.3. Pancreatic Cancer and Normal Control Cohorts

#### 4.3.1. NGS Sample Collection 

Tissue samples were obtained from six PC patients, and six paired controls (stage IIa/IIb, median age of 66.5, range from 55 to 75, male:female of 2:1) using standard protocols of the Inha University Hospital. All samples were formalin-fixed paraffin-embedded and stored at 4 °C.

#### 4.3.2. Microarray Sample Collection 

The Affymetrix-based datasets GSE16515, GSE28735, GSE15471, GSE18670, and GSE41368, were included in the gene expression meta-analysis. After that, we divided the datasets into two groups with similar sample sizes: set A including, GSE16515 and GSE28735 for variable selection purposes; and set B, including GSE15471, GSE18670, and GSE41368 for statistical learning-based validation of the proposed set of novel biomarkers. Dataset GSE19650 was also included for the examination of the multistep PC development.

#### 4.3.3. Tissue-Array Sample Collection

Tissue microarrays containing PC tissues, adjacent normal tissues, and normal tissues (HPan-A150CS-02 and PA1001b) were purchased from US Biomax Inc. (Rockville, MD, USA).

### 4.4. RNA Extraction from Formalin-Fixed Paraffin-Embedded Tissue

Formalin-fixed paraffin-embedded tissue was cut using a razor blade (20 µm), transferred to a 1.5 mL tube, and filled with mineral oil. The tube was incubated at 70 °C for 3 min to melt the paraffin. The sample was then centrifuged for 1 min at 16,000× *g*. The pellet was washed with 1 mL of ethanol and air dried. Next, 150 µL protease K digestion buffer containing 500 µg/mL protease K was added, and the mixture was incubated for 3 h. 1 mL of Trizol was added to the sample and incubated at room temperature for at least 5 min to dissociate nucleoprotein complexes. Subsequently, 0.2 mL chloroform was added, and the mixture was vigorously vortexed for 15 s and centrifuged at 12,000× *g* for 10 min at 4 °C. The aqueous phase was transferred to a fresh tube. Total RNA was precipitated by mixing with an equal volume of isopropyl alcohol at −20 °C for at least 1 h, followed by 10 min of centrifugation at 12,000× *g* at 4 °C. Finally, RNA pellet was washed with 70% ethanol, briefly air-dried, and resuspended in RNase-free water.

### 4.5. RNA Data Processing and Analysis

To construct cDNA libraries, 1 µg of total RNA was used with TruSeq RNA library kit (Illumina, San Diego, CA, USA). The protocol included polyA-selected RNA extraction, RNA fragmentation, random hexamer-primed reverse transcription, and 100-nt paired-end sequencing by Illumina HiSeq2500 (Illumina). The libraries were quantified using qPCR and qualified using an Agilent Technologies 2100 Bioanalyzer (Agilent Technologies, Santa Clara, CA, USA). To estimate the expression level, the RNA-seq reads were mapped to the genome of *Homo sapiens*. The reference genome sequence of *Homo sapiens* and the annotation data were downloaded from the UCSC website (http://genome.uscs.edu). The transcript counts at the gene level were calculated [43]. Data normalization and statistical analysis were then conducted using edgeR. We determined the significant result by adjusting the absolute value of fold change ≥ 2.

### 4.6. Microarray Data Processing and Gene Expression Meta-Analysis

We normalized raw gene expression data using a robust multi-array analysis implemented in the Bioconductor affy package [44]. Database for Annotation, Visualization, and Integrated Discovery (DAVID) version 6.8 was applied to get the Entrez IDs. The Empirical Bayes (ComBat) cross-study normalization method was applied to remove batch effects. The random effects model was chosen as the statistical meta-analysis method based on the result of Cochran’s Q test. The analysis was implemented in the comprehensive web-based tool NetworkAnalyst [45]. 

### 4.7. Immunohistochemistry (IHC) Experiments

The procedures of IHC were carried out as described previously [46]. Two experts independently assessed staining scores. The immunostaining intensity was graded as 0, 1, 2, or 3 for the tissue with stainless cells, <10% stained cells, 10–50% stained cells, and > 50% stained cells, respectively. We excluded the tissues that were not suitable for analysis (e.g., not adenocarcinoma or tissue loss cores). IHC scoring was visualized by Aperio ScanScope digital slide scanners, and analyzed by the vendor’s software (Leica, Wetzlar, Germany). The Mann-Whitney and Wilcoxon matched-pairs signed rank tests were conducted to test two-group differences among PC tissues, adjacent normal tissues, and normal tissues using GraphPad Prism 6 (San Diego, CA, USA) where applicable.

### 4.8. Unsupervised and Supervised Machine Learning Algorithms

#### 4.8.1. Variable Selection

We treated the overexpressed genes coding for secretory and membrane proteins separately because of their distinct clinical roles. Only those coding for secretory proteins and not upregulated in pancreatitis from the compendium were selected for a further classification. 

#### 4.8.2. Data Exploration and Visualization

The principal component analysis (PCA) was incorporated to facilitate data visualization and to detect potential outliers prior to the class assignment analysis using FactoMineR version 1.35 and factoextra version 1.0.4 [47]. A heatmap was also applied to highlight the differences in gene expression between the two comparison groups by means of MetaboAnalyst 3.0 [48]. 

#### 4.8.3. Random Forests Classification Model and Explanation

Classification and Regression Training (caret) package version 6.0.77 was used for data splitting and supervised classification analysis [49]. Set B was divided into the training set and the test set with a ratio of 7:3. A random forests (RF) model using 500 trees was optimized on the training set. The number of variables for splitting at each tree node (mtry) was tuned. Five-time repeated 10-fold cross-validation sampling iteration was used during the training process to select the optimal model. A seed number was given prior to data splitting and model training to achieve reproducible results. We used the explainer local interpretable model-agnostic explanations (LIME) to interpret the rationale behind the black-box RF model’s predictions [50]. 

### 4.9. Correlation Analysis

Pearson’s correlation analysis and Spearman’s rank correlation analysis were conducted for gene expression level and protein expression score, respectively.

### 4.10. Kaplan-Meier Plots and Cox Regression Analysis

The overall survival and disease-free survival were investigated using the Kaplan-Meier method with the log-rank test. We set the high and low gene expression level groups by the median value. The overall survival plot and disease-free survival plot were obtained with the hazard ratios (HR) and the 95% confidence interval information. The whole process was implemented using the web-based tool GEPIA [51]. Additionally, multivariate Cox regression analysis of TCGA PC patients was also extracted using Oncolnc to determine the potential prognostic biomarkers [52]. 

### 4.11. Database Mining, Pathway Enrichment, STRING, and CHAT Analyses

Kyoto Encyclopedia of Genes, Genomes (KEGG) pathway enrichment, and OmicsNet and STRING protein-protein interaction analyses and visualizations were conducted using the significantly overexpressed genes. The Database of Human Pancreatic Cancer (HPCDb) and the Pancreatic Expression Database (PED) were used as resources for literature data mining [53,54]. A public literature review of a reported gene list in PC was also conducted under the guidance of Cancer Hallmarks Analytics Tool (CHAT) [55]. 

### 4.12. Drug-Gene Interaction and miRNA–Gene Interaction for Further Developing Novel Therapeutics

To examine the connection between our membrane protein-coding genes with miRNA, we employed the miRTarBase [56]. The miRTarBase offers data regarding experimentally validated miRNA–target interactions. The Pearson’s correlation coefficient between each candidate and miRNA was also recorded. Subsequently, the drug-gene interaction database was investigated to examine if our genes coded for a drug-metabolizing enzyme or a drug transporter [57]. 

### 4.13. Statistical Significance Level

R statistic 3.4.2 was used to implement the analysis except otherwise stated. A *p*-value of 0.05 was used as the cut-off for significance in statistical tests. False discovery rate (FDR) (Benjamini-Hochberg method), when applicable (e.g., in NGS differential analysis), with a threshold of 0.05, was utilized for all multiple hypothesis tests.

### 4.14. Availability of Data and Materials

NGS data generated and analyzed during the current study are available in the Gene Expression Omnibus (GEO) with the assession code of GSE119224. Other datasets analyzed during the current study are available in the GEO (GSE16515, GSE28735, GSE15471, GSE18670, GSE41368, and GSE19650). All data generated or analyzed during this study are included in this published article and its supplementary information files.

## 5. Conclusions

Recent advances in high-throughput multi–omic technologies along with advanced bioinformatics and machine learning techniques have provided a new paradigm for the discovery of human disease biomarkers [10,46,58,59]. This perspective should remark the continued employment of multi-omic approaches and machine learning techniques under the guidance of big data and biological mechanisms in cancer biomarker research. In view of this, we successfully demonstrated an integrative method to introduce and validate a panel of *LAMC2*, *ANXA2*, *ADAM9*, and *ALPL2* for innovating PC diagnosis, prognosis, and management. Future studies are expected to take a step forward and concentrate on validating biomarkers by integrating multi-omics data in an epidemiological and clinical context-dependent manner.

## Figures and Tables

**Table 1:** Literature- and experiment-based validation of the 23 candidates.

Gene Symbol	Entrez ID	RNA Alteration	Protein Alteration	NGS Results			Meta-Analysis		Neoplasm ^#^ vs. Normalcy	PDAC vs. Neoplasm ^#^
				Log FC	*p*-Value	FDR	cES	*p*-Value		
*AGR2*	10551	↑RT-PCR ^1,2^, ↑SAGE ^1,2^, ↑DM ^1^	↑IHC ^1^, ↑LFQ-MS ^1^, ↑WB ^2^	3.67	1.55 × 10^−15^	7.37 × 10^−13^	1.93	0	↑	X
*GAPDH*	2597	↑DM ^1^, ↑SAGE ^1^, NB ^2^	↑ICAT ^1^, ↑WB ^1,2^, ↓SILAC-TMS ^2^	1.48	6.08 × 10^−4^	1.27 × 10^−2^	1.68	2.02 × 10^−13^	X	X
*LAMC2*	3918	↑DM ^1,2^	↑IHC ^1^	3.91	1.15 × 10^−7^	1.17 × 10^−5^	2.37	0	↑	X
*MMP11*	4320	↑DM ^1^, ↑ISH ^1^, ↑SAGE ^1,2^, ↑NB ^1,2^	↑IHC ^1^, ↑WB ^2^	NA	NA	NA	1.62	7.90 × 10^−14^	X	X
*TAGLN2*	8407	NA	↑SILAC-TMS ^1^, ↑ICAT ^1^	NA	NA	NA	1.73	1.64 × 10^−5^	↑	X
*ADAM9*	8754	↑DM ^1,2^, ↑RT-PCR ^1,2^	↑IHC ^1^	NA	NA	NA	1.71	1.41 × 10^−5^	X	X
*ANXA2*	302	↑DM ^1^	↑IHC ^1^, ↑ICAT ^1^, ↑WB ^1,2^, ↑MS ^1^, ↑TDE ^1^, ↑LFQ-MS ^1^	1.16	2.26 × 10^−3^	3.35 × 10^−2^	1.63	8.33 × 10^−9^	X	X
*APLP2*	334	↑DM ^1,2^	↑SILAC-TMS ^1,2^, ↑SILAC ^2^	NA	NA	NA	1.53	1.11 × 10^−5^	X	X
*CDH3*	1001	↑DM ^1,2^, ↑RT-PCR ^1,2^	↑IHC ^1^	2.72	8.70 × 10^−6^	4.50 × 10^−4^	2.02	0	X	↑
*MSLN*	10232	↓DM ^1^, ↑DM ^1,2^, ↑RT-PCR ^1,2^, ↑SAGE ^1,2^, ↑ISH ^1^	↑IHC ^1^	5.21	1.89 × 10^−15^	8.77 × 10^−13^	2.03	4.88 × 10^−4^	↑	X
*SERPINB5*	5268	↑DM ^1,2^, ↑NB ^2^	↑IHC ^1,2^, ↑SILAC-TMS ^1^	8.35	6.54 × 10^−30^	2.96 × 10^−26^	1.96	0	↑	X
*CD82*	3732	↑NB ^1^, ↑ISH ^1^, ↑DM ^1^	↑IHC ^1^	1.39	4.55 × 10^−4^	1.03 × 10^−2^	1.62	7.90 × 10^−14^	X	X
*CLDN18*	51208	↑DM ^1^	↑IHC ^1^	2.57	9.43 × 10^−5^	2.97 × 10^−3^	1.64	7.60 × 10^−9^	↑	X
*EPHA2*	1969	↑DM ^1,2^	↑SILAC-TMS ^1^, ↑WB ^2^	1.69	4.95 × 10^−5^	1.78 × 10^−3^	1.56	2.99 × 10^−13^	X	↑
*EZR*	7430	NA	↑IHC ^1^, SILAC-TMS ^1^	NA	NA	NA	1.68	0	↑	X
*FXYD3*	5349	↑DM ^1,2^, ↑SAGE ^1^, ↑NB ^1^, ↑ISH ^1^	NA	4.71	4.03 × 10^−21^	5.21 × 10^−18^	1.91	0	↑	X
*GPRC5A*	9052	↑DM ^1,2^, ↑SAGE ^1,2^	↑IHC ^1^	3.98	2.08 × 10^−8^	2.61 × 10^−6^	1.91	1.25 × 10^−8^	↑	↑
*ITGA2*	3673	↑DM ^1,2^	↑IHC ^1^, ↑WB ^1^	2.94	1.20 × 10^−6^	8.72 × 10^−5^	2.04	2.02 × 10^−13^	↑	X
*ITGB6*	3694	↑DM ^1^	↑IHC ^1^	2.56	4.55 × 10^−6^	2.71 × 10^−4^	1.79	2.78 × 10^−8^	X	X
*MET*	4233	↑DM ^1^, ↑RT-PCR ^1,2^	NA	NA	NA	NA	1.90	1.97 × 10^−7^	X	X
*MST1R*	4486	↑DM ^1,2^, ↑RT-PCR ^2^	↑IHC ^1^, ↑WB ^2^	2.84	9.03 × 10^−7^	6.81 × 10^−5^	2.06	0	↑	X
*NQO1*	1728	↑DM ^1,2^	↑IHC ^1^, ↑WB ^2^	3.71	1.88 × 10^−9^	3.04 × 10^−7^	2.15	1.63 × 10^−5^	↑	X
*SLC2A1*	6513	↑DM ^1,2^	↑IHC ^1^	3.28	6.34 × 10^−10^	1.16 × 10^−7^	2.03	0	↑	↑

^#^ Data obtained from the dataset GSE19650. ^1^ Evidence in PDAC tissue. ^2^ Evidence in cancer cell lines. Abbreviations: cES: combined effect size; DM: DNA microarray; FDR: false discovery rate; ICAT: isotope-coded affinity tag; IHC: immunohistochemistry; IPMN: intraductal papillary mucinous neoplasia; ISH: in situ hybridization; LFQ-MS: label-free quantitation mass spectrometry; NB: northern blot; NGS: next-generation sequencing; PDAC: pancreatic ductal adenocarcinoma; RT-PCR: reverse transcription-polymerase chain reaction; SAGE: serial analysis of gene expression; SILAC-TMS: stable isotope labeling by/with amino acids in cell culture-tandem mass spectrometry; TDE: two dimensional electrophoresis; WB: western blot; NA: Not applicable/available.

**Table 2:** Combined effect sizes, Cox regression analysis, and alterations at the mRNA and protein level of the 23 candidates.

Gene Symbol	Entrez ID	Cox Regression	
		Cox Coefficient	FDR
*AGR2*	10551	0.05	0.76
*GAPDH*	2597	0.21	0.19
*LAMC2*	3918	0.42	0.02
*MMP11*	4320	0.17	0.28
*TAGLN2*	8407	0.19	0.22
*ADAM9*	8754	0.41	0.02
*ANXA2*	302	0.32	0.04
*APLP2*	334	0.23	0.14
*CDH3*	1001	0.37	0.03
*MSLN*	10232	0.26	0.10
*SERPINB5*	5268	0.38	0.02
*CD82*	3732	0.11	0.51
*CLDN18*	51208	0.09	0.63
*EPHA2*	1969	0.33	0.05
*EZR*	7430	0.28	0.08
*FXYD3*	5349	0.17	0.30
*GPRC5A*	9052	0.37	0.03
*ITGA2*	3673	0.41	0.02
*ITGB6*	3694	0.49	0.01
*MET*	4233	0.66	0.00
*MST1R*	4486	0.22	0.17
*NQO1*	1728	0.14	0.43
*SLC2A1*	6513	0.27	0.08

**Table 3:** Drug–gene and miRNA–gene interactions of 18 membrane proteins.

Gene Symbol	miRNA–Gene Interaction				Druggable Genome	Drug–Gene Interaction ^3^
	miRNA ^1^ That Targets the Gene	Validation Methods		Expression Profile in PC (Correlation Coefficient ^2^)		
		Strong Evidence	Less Strong Evidence			
*ADAM9*	hsa-miR-126-3p	RA, WB, qPCR	MA	0.315	Yes	Ilomastat
	hsa-miR-33a-5p	RA, WB, qPCR		0.373		
	hsa-miR-125a-5p	qPCR		0.320		
*ANXA2*	hsa-miR-155-5p	RA, WB, qPCR	MA	−0.599	Yes	NA
	hsa-miR-206	RA, WB, qPCR		−0.312		
*APLP2*	NA				Yes	NA
*CDH3*	NA				Yes	NA
*MSLN*	hsa-miR-21-5p	RA		0.715	Yes	Amatuximab
*SERPINB5*	hsa-miR-21-5p	RA, WB, qPCR	MA, NGS	0.709	Yes	NA
	hsa-miR-103a-3p	RA, WB, qPCR	MA, NGS	0.501		
*CD82*	NA				Yes	NA
*CLDN18*	NA				Yes	Claudiximab
*EPHA2*	NA				Yes	Dasatinib, Dorsomorphin, Regorafenib, Vandetanib
*EZR*	hsa-miR-183-5p	RA, WB, qPCR		0.424	NA	NA
	hsa-miR-204-5p	RA, WB, qPCR	MA	−0.542		
	hsa-miR-205-5p	RA	MA, NGS	0.358		
*FXYD3*	NA				No	NA
*GPRC5A*	hsa-miR-103a-3p	RA, WB, qPCR	NGS	0.514	Yes	NA
*ITGA2*	hsa-miR-16-5p	qPCR	NGS, pSILAC	0.509	Yes	Abciximab, CHEMBL36326, Eptifibatide, Tirofiban, Vatelizumab
*ITGB6*	NA				Yes	DI17E6, Intetumumab, STX-100
*MET*	hsa-miR-34c-5p	RA, WB, qPCR	MA, NGS	0.337	Yes	ARRY-300, ABT-700, AMG-337, AMG-208, Amuvatinib, Altiratinib, Amoxicillin, Alectinib, BMS-698769, BMS-777607, BMS-794833, BMS-817378, BPI-9016, Crizotinib, Clofibrate, Cabozantinib, Cabozantinib S-Malate, Capmatinib, Crenolanib, Emibetuzumab, EMD-1204831, Foretinib, Golvatinib, JNJ-38877605, LY-2875358, MK-8033, MGCD-265, Merestinib, MK-2461, Onartuzumab, PF-04217903, PHA-665752, Pyrazinamide, Rilonacept, SGX-523, Savolitinib, Tepotinib, Tivantinib, Tanespimycin, SAR-125844, TAS-115
	hsa-miR-199a-3p	RA, WB, qPCR	MA	0.365		
	hsa-miR-34a-5p	RA, WB, qPCR		0.478		
	hsa-miR-23b-3p	RA, WB, qPCR		0.447		
	hsa-miR-27a-3p	RA, WB, qPCR		0.444		
	hsa-miR-27b-3p	RA, WB, qPCR		0.458		
	hsa-miR-31-5p	RA, qPCR		0.594		
	hsa-miR-34a-3p	WB		0.380		
*MST1R*	NA				Yes	BMS-777607, Foretinib, MGCD-265, MK-2461, MK-8033
*NQO1*	NA				Yes	Apaziquone, Dicumarol, Vatiquinone
*SLC2A1*	hsa-miR-22-3p	RA, WB, qPCR	NGSs	0.331	Yes	NA

^1^ Only miRNAs that have strong evidence were included, ^2^ *p*-value of the correlation coefficient was less than 0.05. ^3^ Only defined interactions were included. Abbreviation: MA: microarray; NGS: next-generation sequencing; pSILAC: pulsed stable isotope labeling by amino acids in cell culture; qPCR: quantitative polymerase chain reaction; RA: reporter assay; WB: western blot.

## Supplementary Materials

The following are available online at http://www.mdpi.com/2072-6694/11/2/155/s1. Figure S1: Overall survival and disease-free survival plots of the 23 candidates. Figure S2: Boxplots of the remaining nine secretory protein-coding genes; *p*-value < 0.0001 in all analyses. Figure S3: LIME of four representative cases. Figure S4: Spearman’s pairwise correlation analysis for *ANXA2*, *ADAM9*, and *LAMC2*. Figure S5: Protein expression level of three promising candidates in eight matched PC and normal samples. Figure S6: Protein expression level of APLP2 in 64 matched PC and normal samples. *p*-value < 0.05. Figure S7: Protein-protein interaction network of 397 upregulated genes in the meta-analysis. Table S1: Characteristics of the included microarray gene expression datasets. Table S2: Characteristics of the cohort used for assessing the expression of LAMC2, ADAM9, and ANXA2. Table S3: Characteristics of the cohort used for assessing the expression of APLP2. Table S4: Significantly enriched pathways of 397 upregulated genes from the meta-analysis of five datasets. Table S5: Summary of the CHAT analysis.
