# Supplementary material for: An Integrative Data Mining and Omics-Based Translational Model for the Identification and Validation of Oncogenic Biomarkers of Pancreatic Cancer
Source: Cancers (Basel). 2019 Jan 29;11(2):155. doi: 10.3390/cancers11020155 (PMC6407035; doi:10.3390/cancers11020155)
Supplement: Supplementary file 1 [file cancers-11-00155-s001.pdf]

Supplementary Materials: An Integrative Data Mining and Omics-Based Translational Model for the Identification and Validation of Oncogenic Biomarkers of Pancreatic Cancer

Nguyen Phuoc Long, Kyung Hee Jung, Nguyen Hoang Anh, Hong Hua Yan, Tran Diem Nghi, Seongoh Park, Sang Jun Yoon, Jung Eun Min, Hyung Min Kim, Joo Han Lim, Joon Mee Kim, Johan Lim, Sanghyuk Lee, Soon-Sun Hong and Sung Won Kwon

AGR2

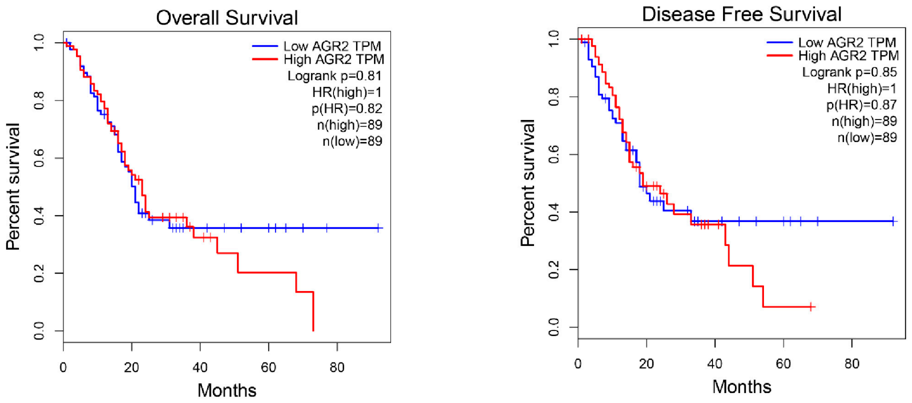

GAPDH

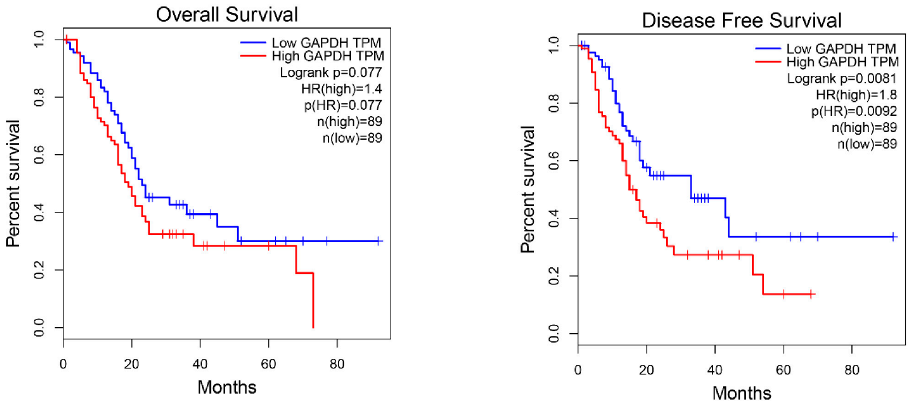

LAMC2

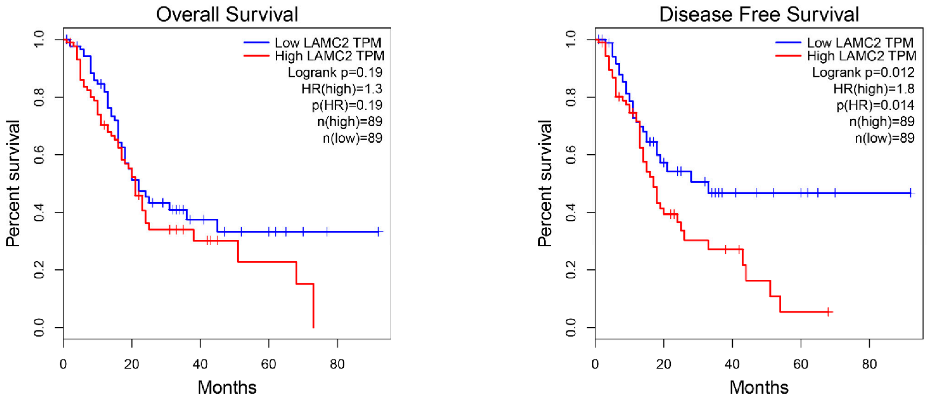

MMP11

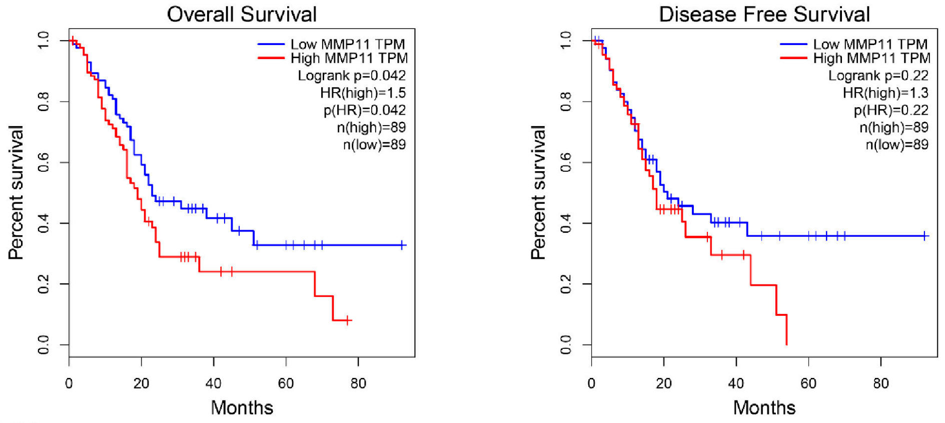

TAGLN2

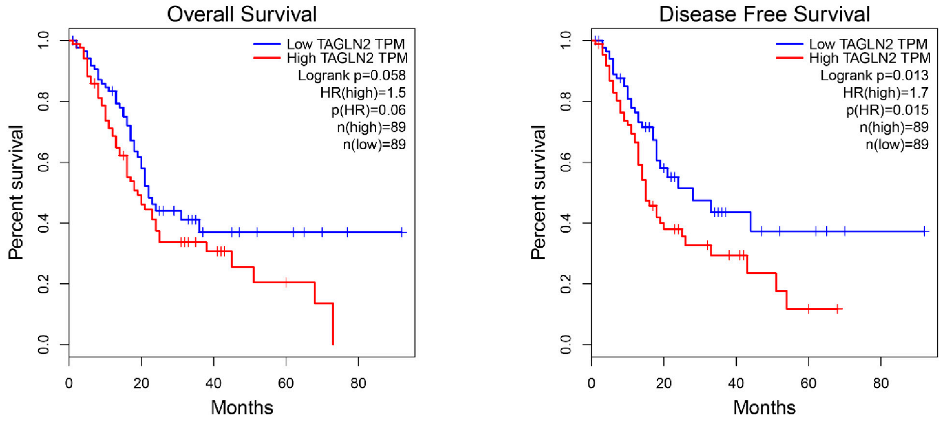

ADAM9

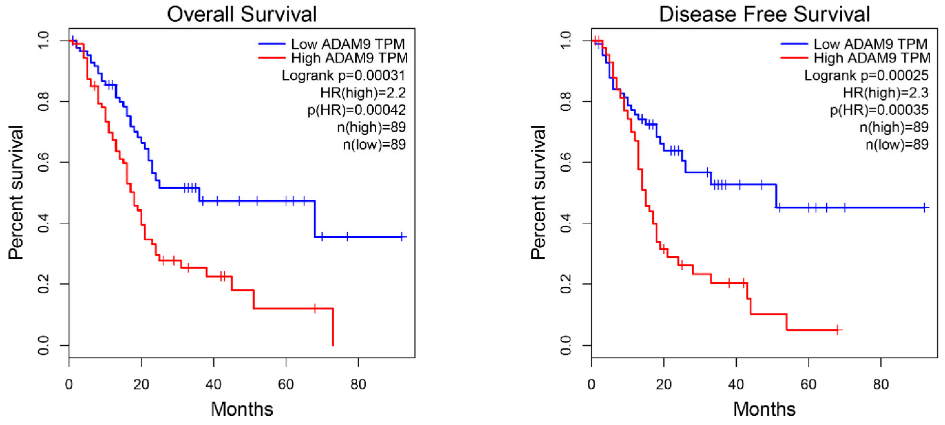

ANXA2

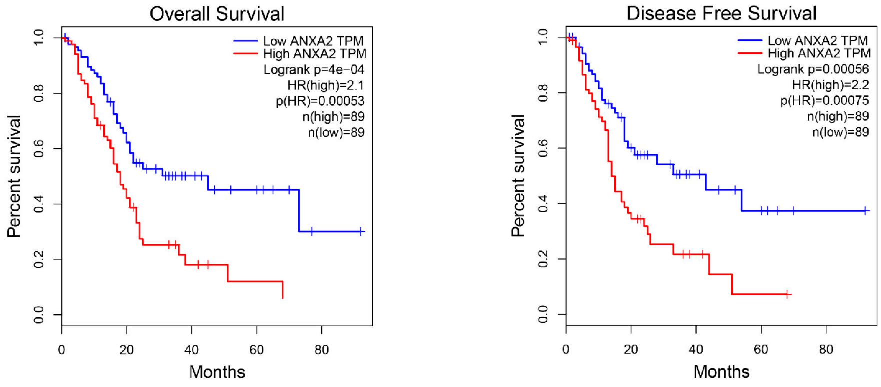

APLP2

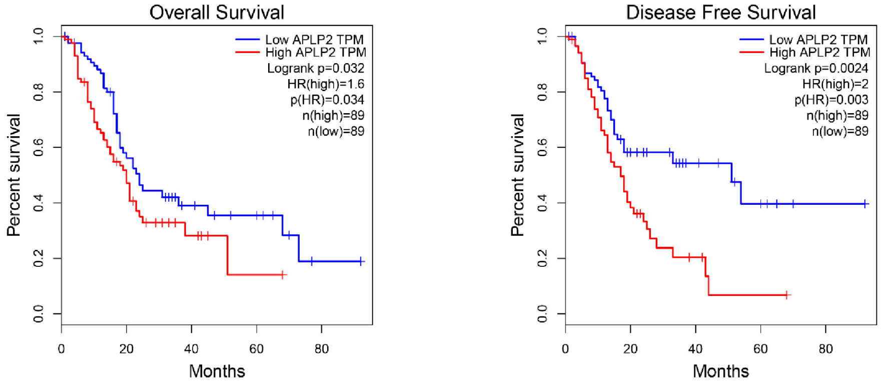

CDH3

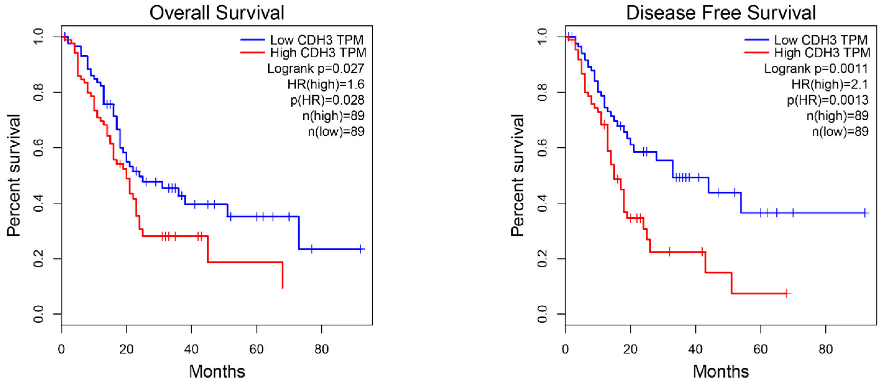

MSLN

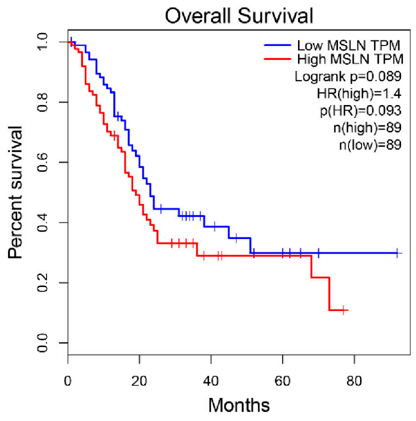

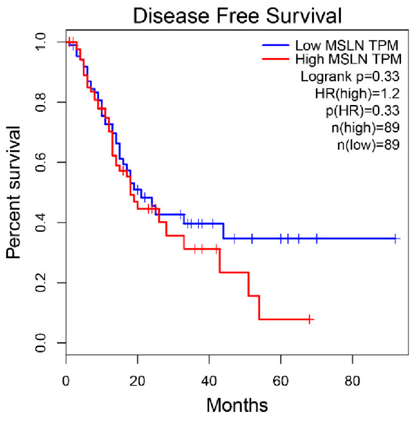

SERPINB5

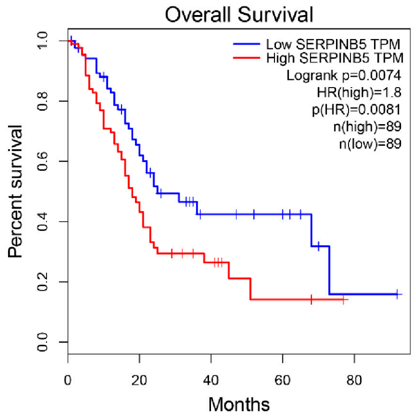

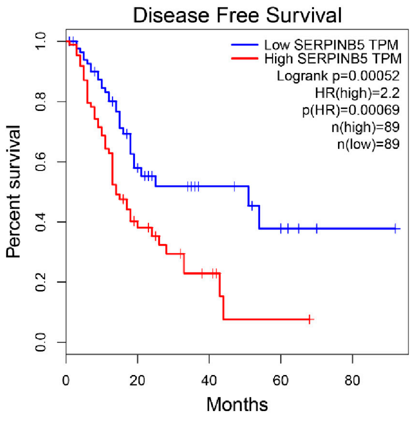

CD82

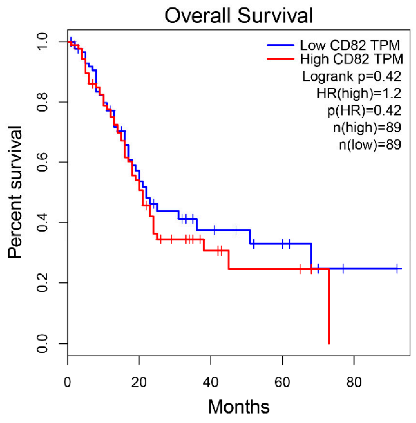

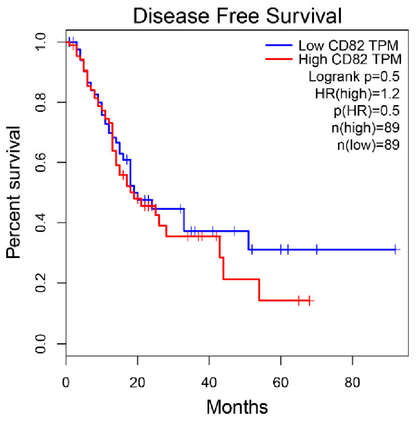

CLDN18

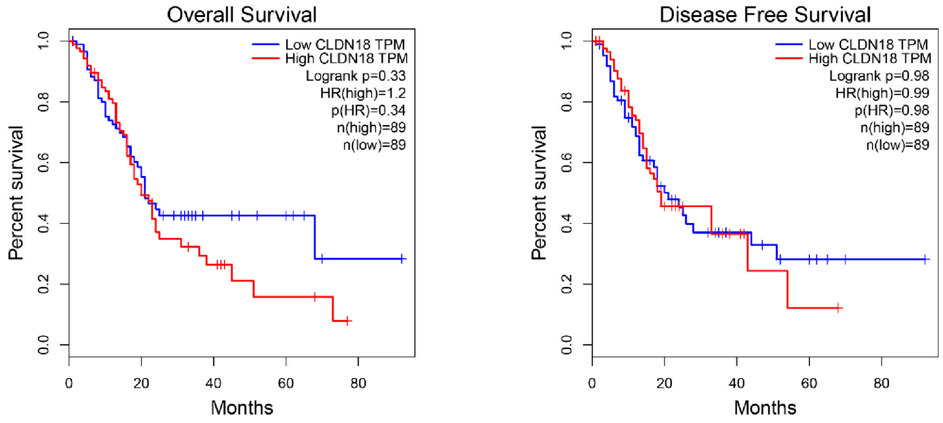

EPHA2

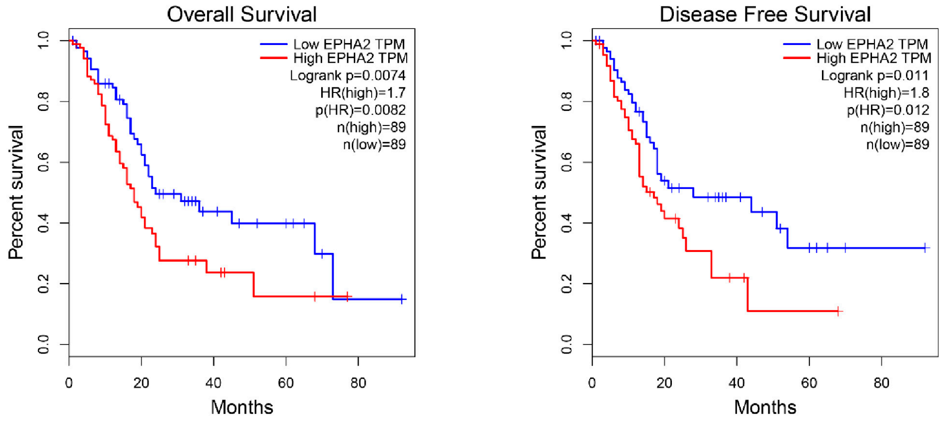

EZR

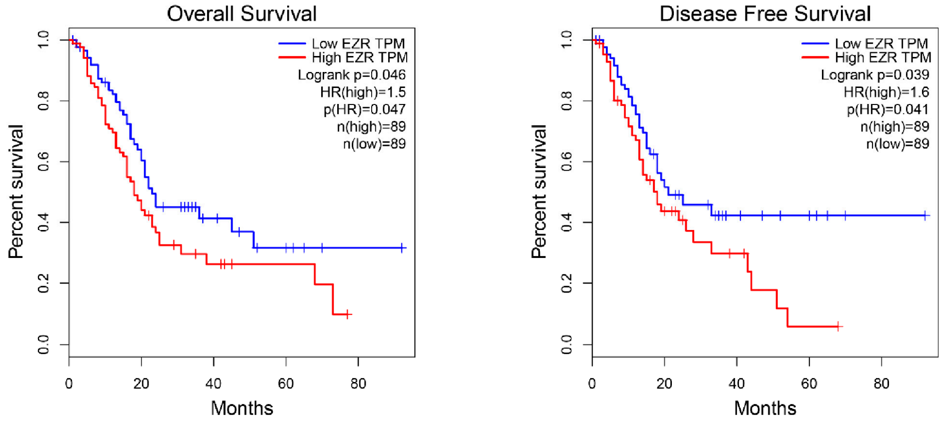

FXYD3

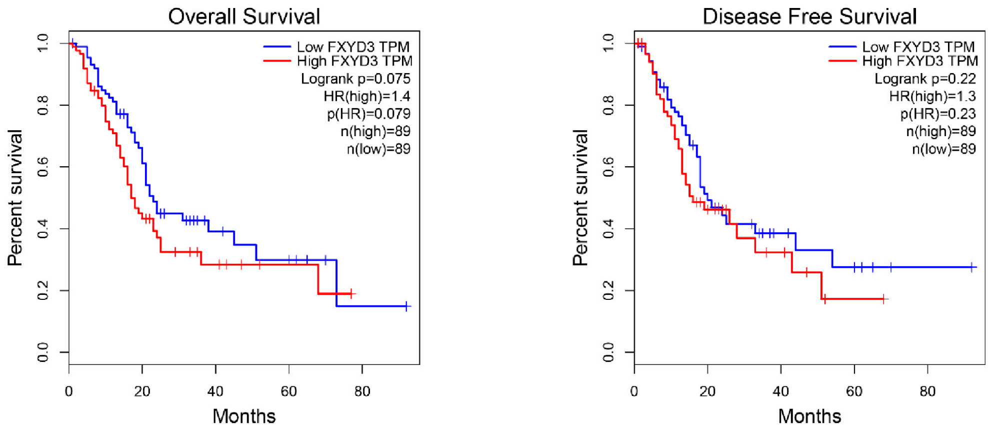

GPRC5A

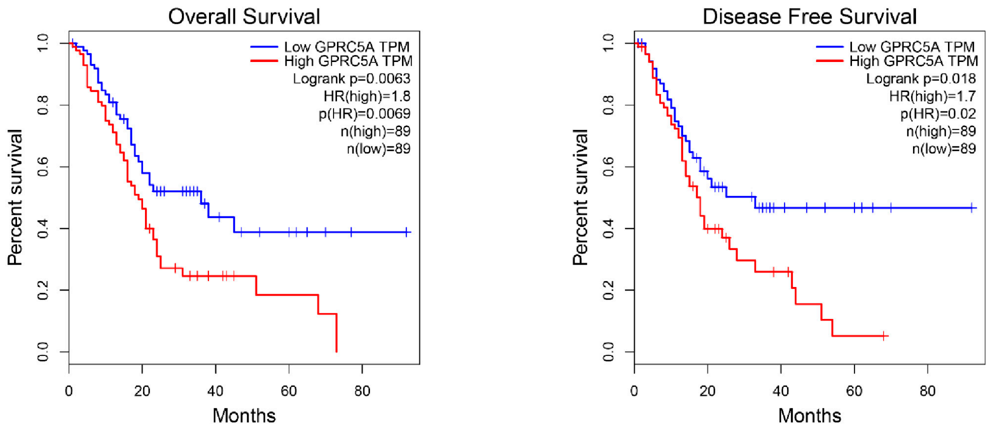

ITGA2

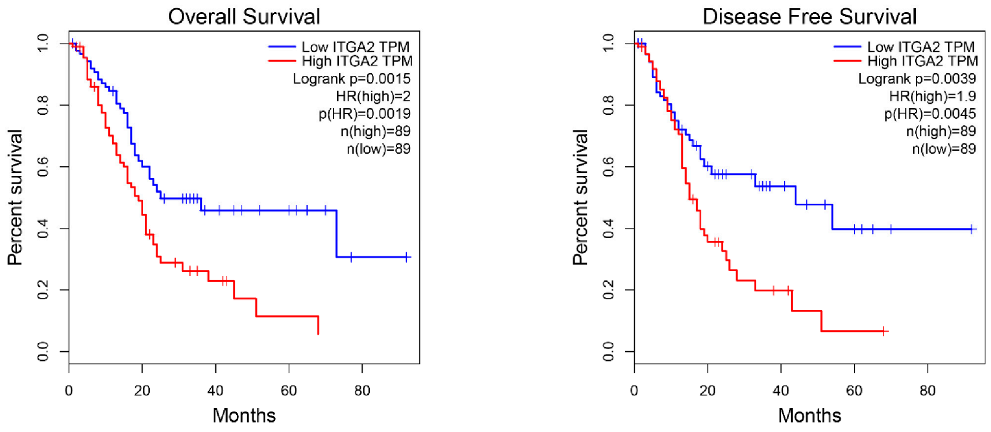

ITGB6

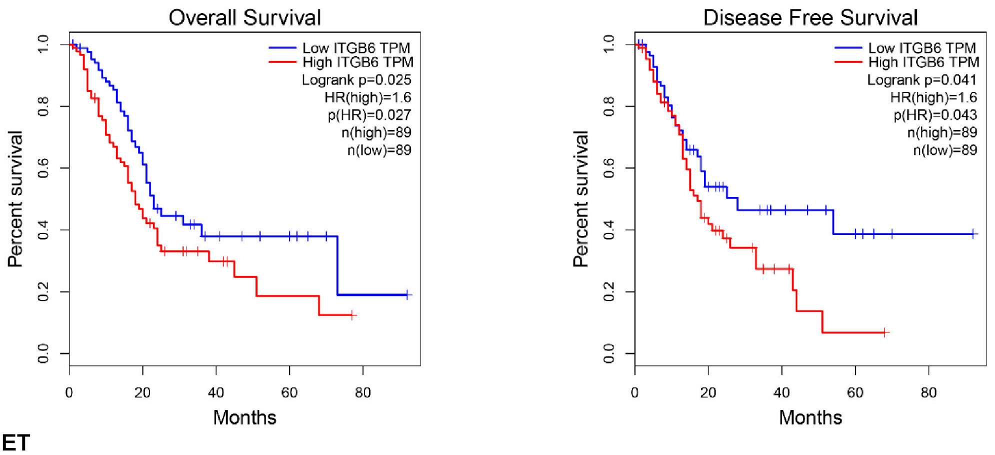

MET

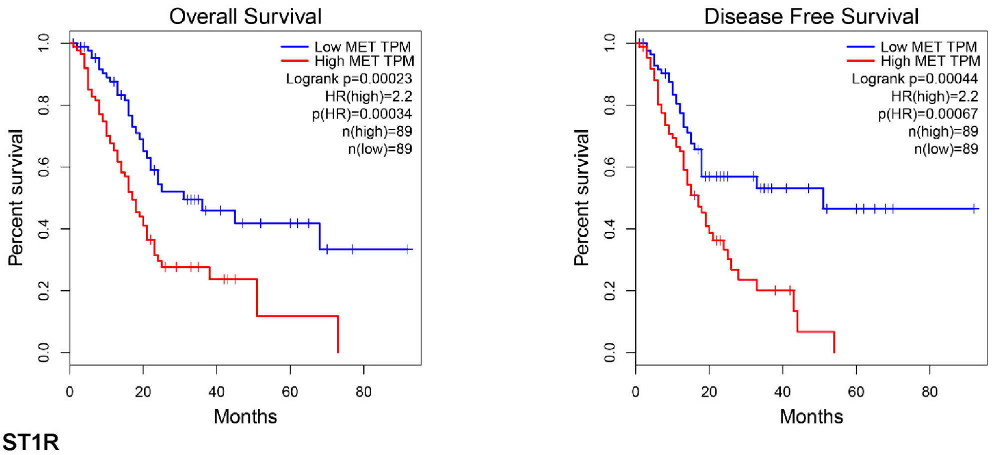

MST1R

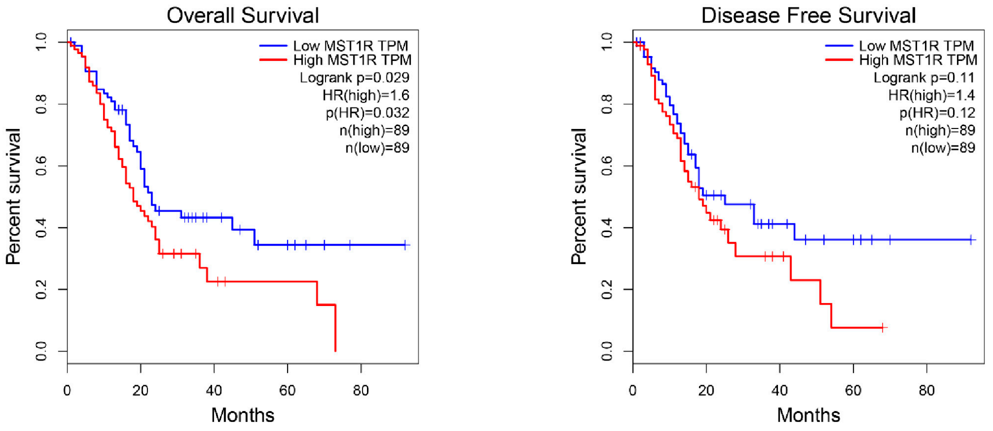

NQO1

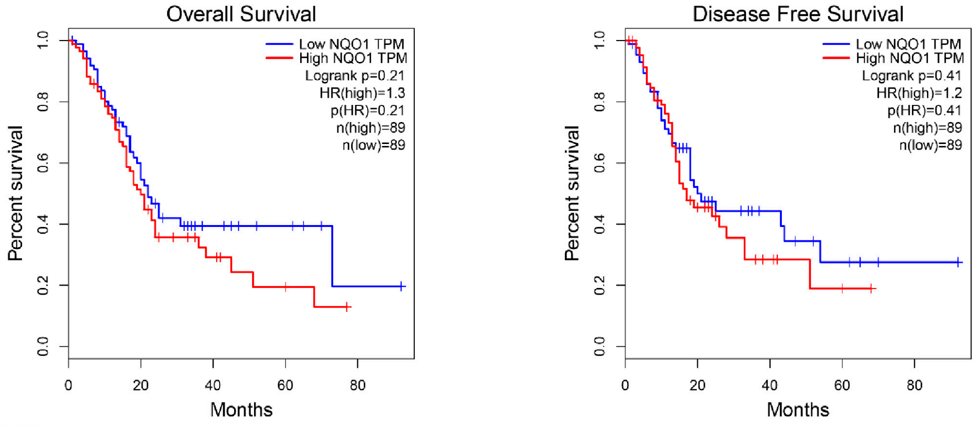

SLC2A1

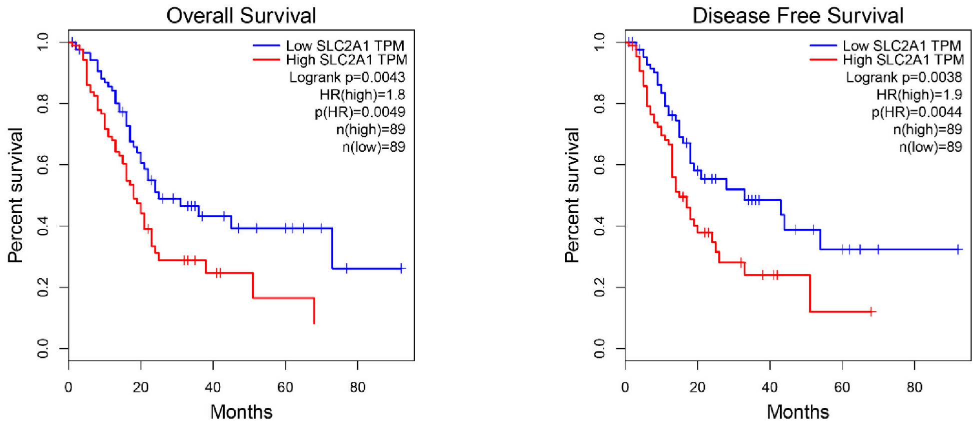

Figure S1. Overall survival and disease-free survival plots of the 23 candidates.

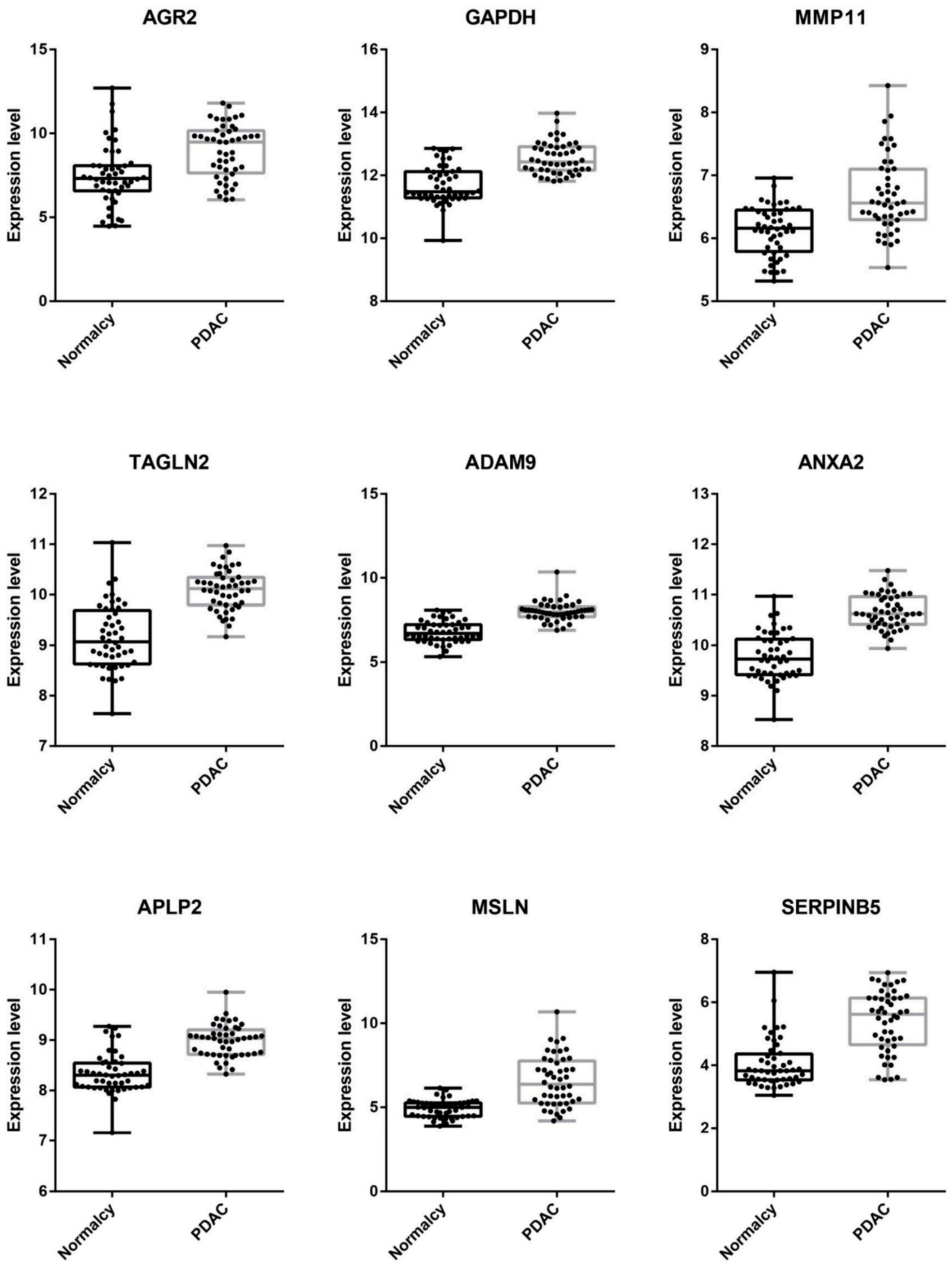

Figure S2. Boxplots of the remaining nine secretory protein-coding genes. p value < 0.0001 was considered statistically significant in all analyses.

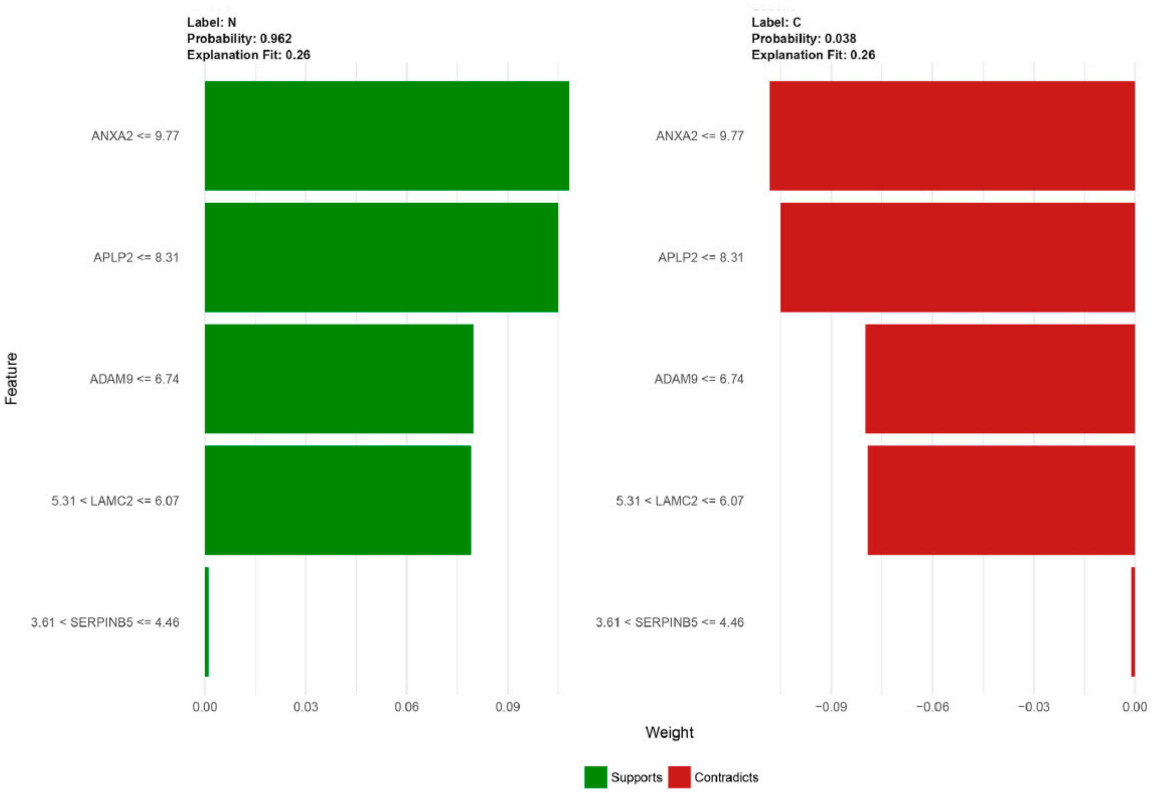

(Case 1)

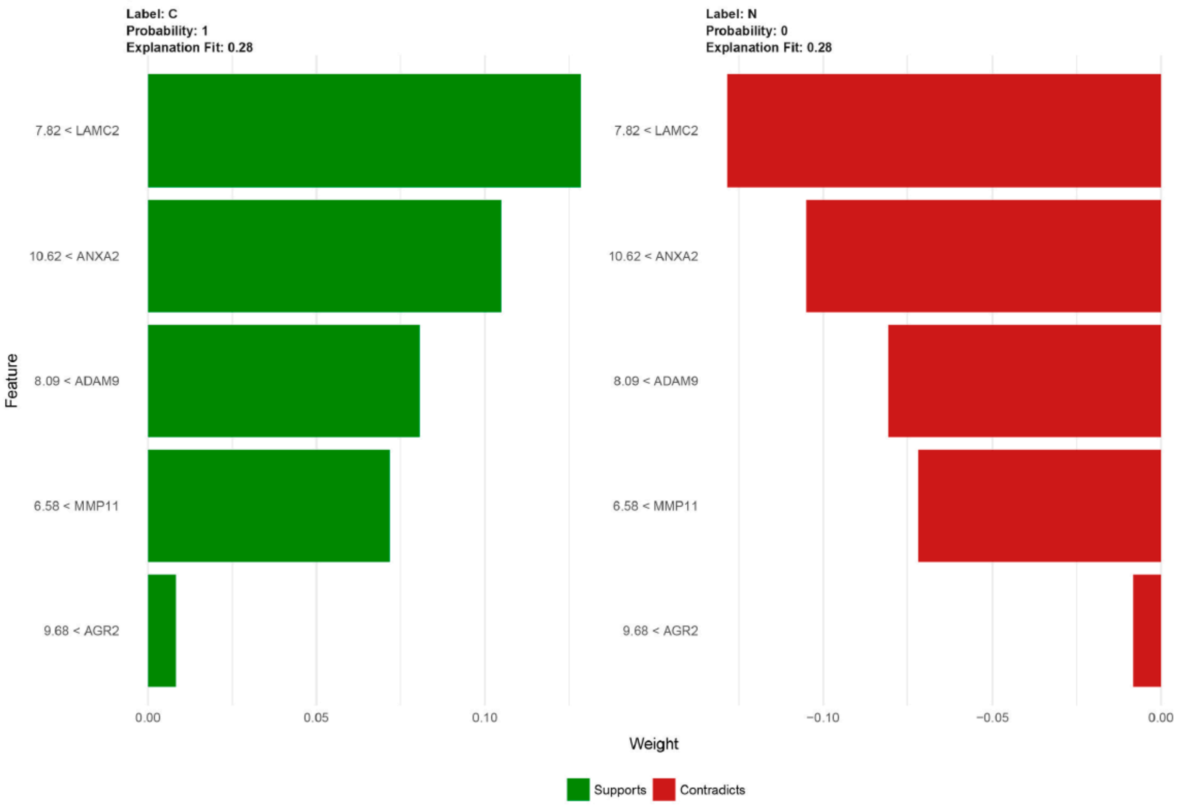

(Case 17)

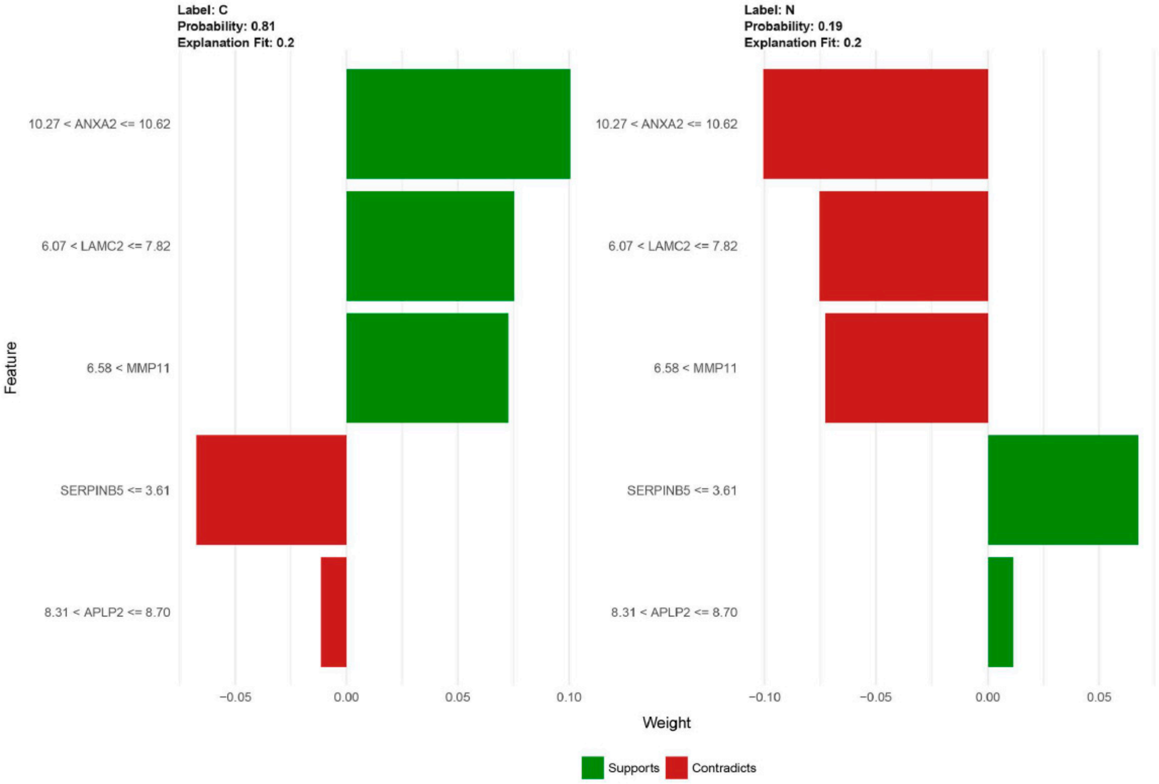

(Case 11)

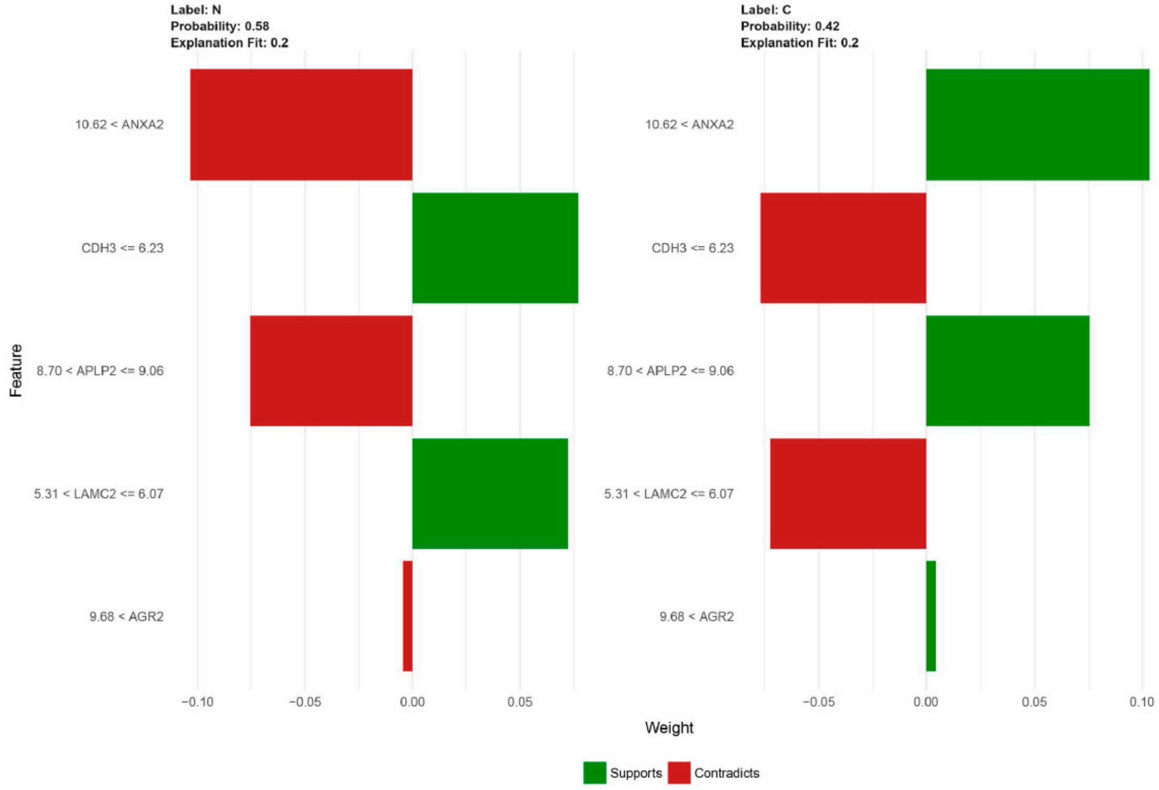

(Case 4)

Figure S3. LIME of four representative cases. **Case 1** was certainly predicted as normal tissue based on the rules: ANXA2 ≤ 9.77, APLP2 ≤ 8.31, ADAM9 ≤ 6.74, 5.31 <LAMC2 ≤ 6.07, and 3.61 < SERPINB5 ≤ 4.46, reaching the probability of 0.962. **Case 17** was certainly predicted as PC based on the rules: LAMC2 > 7.82, ANXA2 > 10.62, ADAM9 > 8.09, MMP11 > 6.58, and AGR2 > 9.68, reaching the probability of 1.00. **Case 11** was misclassified from normal case into PC group, likely due to the overexpression of ANXA2 (ANXA2 > 10.27), MMP11 (MMP11 > 6.58), and LAMC2 (LAMC2 > 6.07). **Case 4** was accurately diagnosed normal with the probability of only 0.58 due to the very high expression level of ANXA2(ANXA2 > 10.62) and APLP2 (APLP2 > 8.70).

a

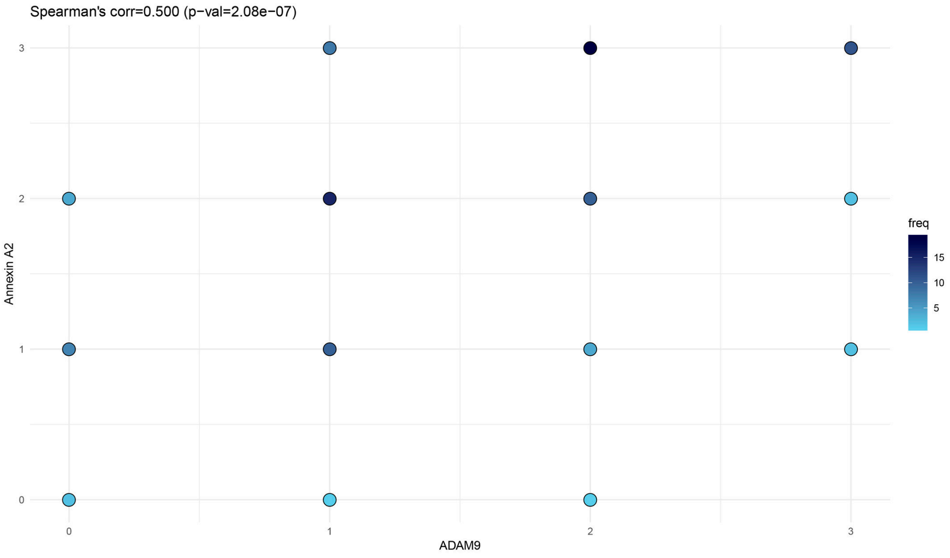

b

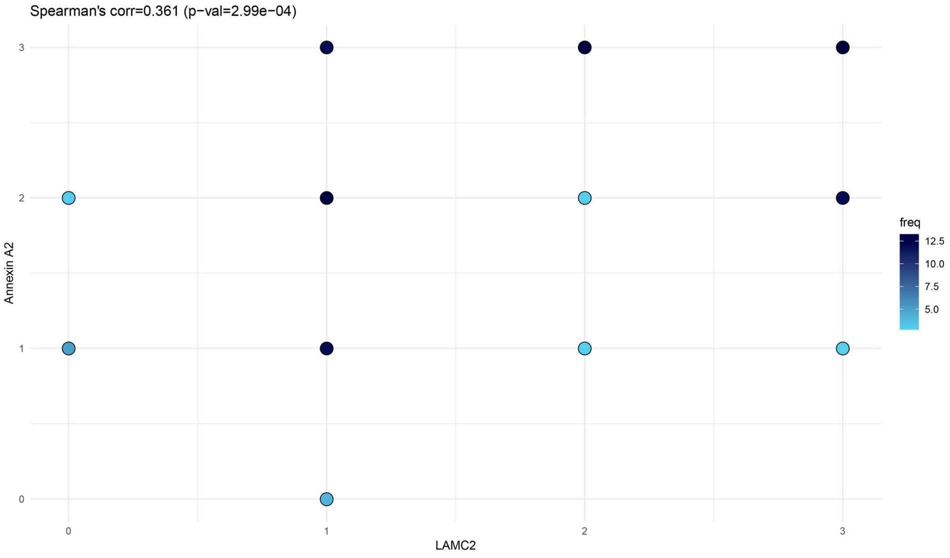

Figure S4. Spearman's pairwise correlation analysis for ANXA2, ADAM9, and LAMC2. (**a**). Spearman's pairwise correlation between Annexin A2 and ADAM9. (**b**). Spearman's pairwise correlation between Annexin A2 and LAMC2.

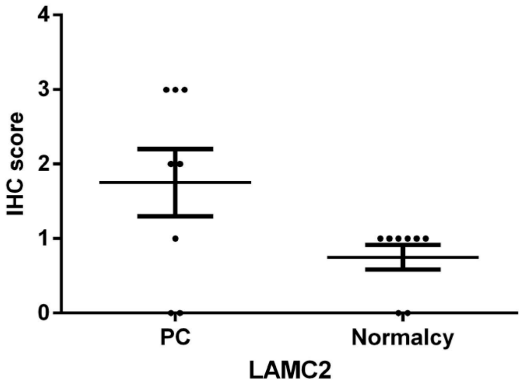

(**a**)

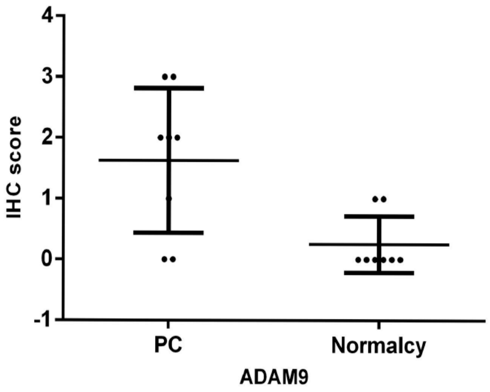

(**b**)

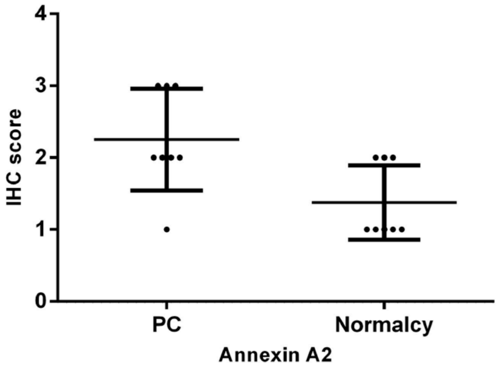

(**c**)

Figure S5. Protein expression level of three promising candidates in eight matched PC and normal samples. (**a**) Protein expression level of LAMC2. (**b**) Protein expression level of ADAM9. (**c**) Protein expression level of Annexin A2.

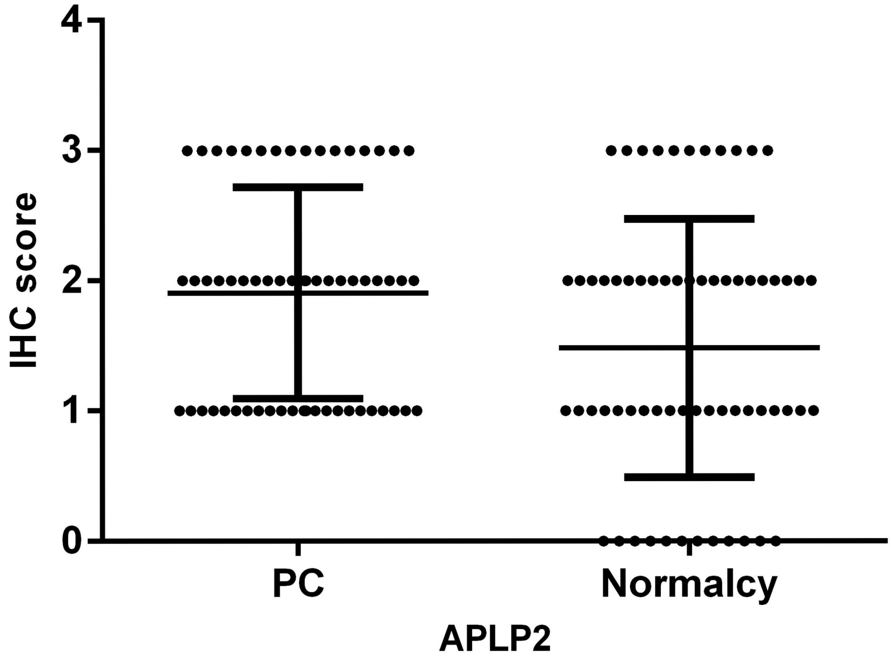

Figure S6. Protein expression level of APLP2 in 64 matched PC and normal samples.

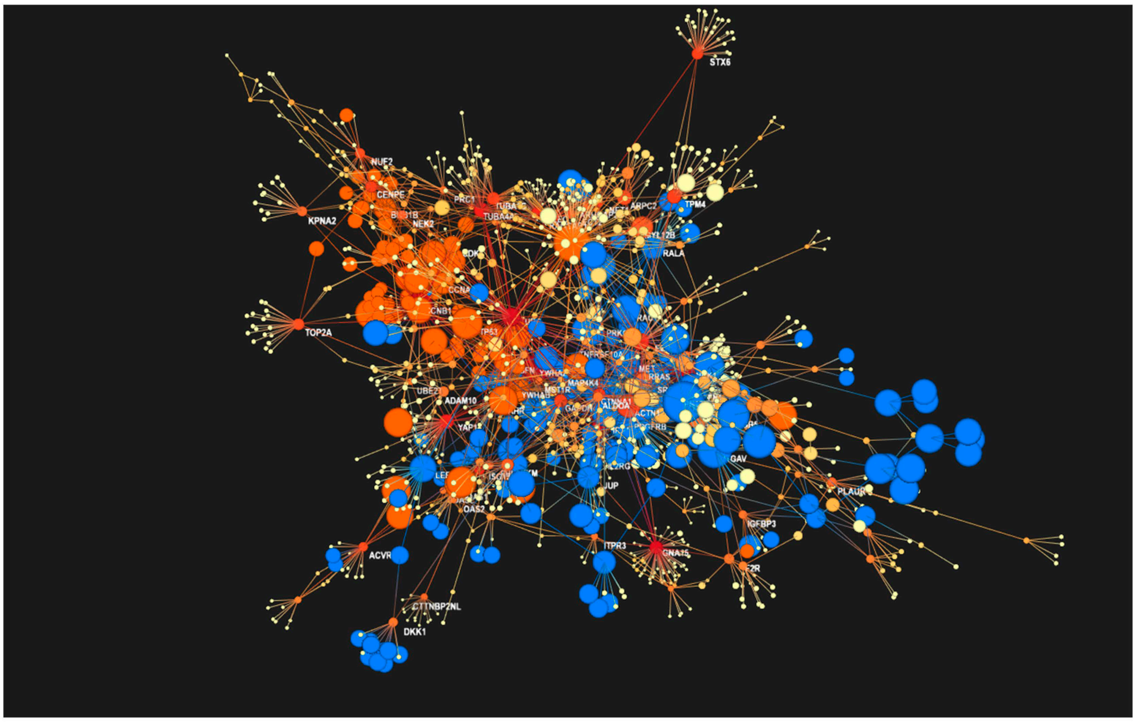

Figure S7. Protein-protein interaction network of 397 upregulated genes in the meta-analysis.

Table S1. Characteristics of the included microarray gene expression datasets.

Author	Data Set	Year	Platform	Country	Samples		
					Normalcy	Cancer	Neoplasm
Frampton AE et al.	GSE41368	2013	1.0 ST	Italy	6	6	
Zhang G et al.	GSE28735	2012	1.0 ST	USA	45	45	
Sergeant G et al.	GSE18670	2012	U133 Plus 2.0	Belgium	6	6	
Pei H et al.	GSE16515	2009	U133 Plus 2.0	USA	16	36	
Badea L et al.	GSE15471	2009	U133 Plus 2.0	Romania	36	36	
Hiraoka N et al.	GSE19650 *	2010	U133 Plus 2.0	Japan	7	3	12

* This data set was used for single analysis.

Table S2. Characteristics of the cohort used for assessing the expression of LAMC2, ADAM9, and ANXA2.

Position	No.	Age	Sex	Organ/Anatomic Site	Pathology Diagnosis	TNM	Grade	Stage	Type	Tissue ID.
A1	1	38	F	Pancreas	Duct adenocarcinoma	T2N0M0	1		Malignant	Dpa090259
A2	2	38	F	Pancreas	Duct adenocarcinoma	T2N0M0	1		Malignant	Dpa090259
A3	3	74	M	Pancreas	Duct adenocarcinoma (fibrous tissue and blood vessel)	T3N0M0	-		Malignant	Dpa080146
A4	4	74	M	Pancreas	Duct adenocarcinoma (sparse)	T3N0M0	-		Malignant	Dpa080146
A5	5	47	M	Pancreas	Duct adenocarcinoma	T2N0M0	1		Malignant	Dpa090197
A6	6	47	M	Pancreas	Duct adenocarcinoma	T2N0M0	1		Malignant	Dpa090197
A7	7	40	M	Pancreas	Duct adenocarcinoma	T2N1M0	1		Malignant	Dpa090186
A8	8	40	M	Pancreas	Duct adenocarcinoma	T2N1M0	1		Malignant	Dpa090186
A9	9	54	M	Pancreas	Duct adenocarcinoma	T2N0M0	1		Malignant	Dpa090177
A10	10	54	M	Pancreas	Duct adenocarcinoma	T2N0M0	1		Malignant	Dpa090177
B1	11	64	F	Pancreas	Duct adenocarcinoma	T2N0M0	1		Malignant	Dpa090166
B2	12	64	F	Pancreas	Duct adenocarcinoma	T2N0M0	1		Malignant	Dpa090166
B3	13	80	M	Pancreas	Duct adenocarcinoma	T2N0M0	2		Malignant	Dpa090119
B4	14	80	M	Pancreas	Duct adenocarcinoma	T2N0M0	2		Malignant	Dpa090119
B5	15	68	F	Pancreas	Duct adenocarcinoma	T3N0M0	2		Malignant	Dpa041572
B6	16	68	F	Pancreas	Duct adenocarcinoma	T3N0M0	2		Malignant	Dpa041572
B7	17	62	F	Pancreas	Duct adenocarcinoma	T3N1M0	2		Malignant	Dpa100207
B8	18	62	F	Pancreas	Duct adenocarcinoma	T3N1M0	-		Malignant	Dpa100207
B9	19	49	M	Pancreas	Duct adenocarcinoma	T3N0M0	2		Malignant	Dpa040571
B10	20	49	M	Pancreas	Duct adenocarcinoma	T3N0M0	2		Malignant	Dpa040571
C1	21	56	F	Pancreas	Duct adenocarcinoma (sparse)	T2N0M0	1		Malignant	Dpa080285
C2	22	56	F	Pancreas	Duct adenocarcinoma	T2N0M0	1		Malignant	Dpa080285
C3	23	61	M	Pancreas	Duct adenocarcinoma	T3N0M1	2		Malignant	Dpa040165
C4	24	61	M	Pancreas	Duct adenocarcinoma	T3N0M1	2		Malignant	Dpa040165
C5	25	52	M	Pancreas	Duct adenocarcinoma	T2N0M0	3		Malignant	Dpa040753
C6	26	52	M	Pancreas	Duct adenocarcinoma	T2N0M0	3		Malignant	Dpa040753

C7	27	46	F	Pancreas	Duct adenocarcinoma	T2N0M0	2	Malignant	Dpa080281
C8	28	46	F	Pancreas	Duct adenocarcinoma	T2N0M0	2	Malignant	Dpa080281
C9	29	64	M	Pancreas	Duct adenocarcinoma	T3N0M0	2–3	Malignant	Dpa031824
C10	30	64	M	Pancreas	Duct adenocarcinoma	T3N0M0	2–3	Malignant	Dpa031824
D1	31	48	F	Pancreas	Duct adenocarcinoma	T3N0M0	2	Malignant	Dpa030776
D2	32	48	F	Pancreas	Duct adenocarcinoma	T3N0M0	2	Malignant	Dpa030776
D3	33	76	F	Pancreas	Duct adenocarcinoma	T4N0M0	2	Malignant	Dpa030468
D4	34	76	F	Pancreas	Duct adenocarcinoma with necrosis	T4N0M0	2	Malignant	Dpa030468
D5	35	65	M	Pancreas	Duct adenocarcinoma	T3N0M0	2	Malignant	Dpa031922
D6	36	65	M	Pancreas	Duct adenocarcinoma	T3N0M0	2	Malignant	Dpa031922
D7	37	64	M	Pancreas	Duct adenocarcinoma	T3N0M0	2	Malignant	Dpa040561
D8	38	64	M	Pancreas	Duct adenocarcinoma	T3N0M0	2	Malignant	Dpa040561
D9	39	66	F	Pancreas	Duct adenocarcinoma	T2N0M0	3	Malignant	Dpa090156
D10	40	66	F	Pancreas	Duct adenocarcinoma	T2N0M0	3	Malignant	Dpa090156
E1	41	65	M	Pancreas	Duct adenocarcinoma with necrosis	T3N1M0	2	Malignant	Dpa080082
E2	42	65	M	Pancreas	Duct adenocarcinoma	T3N1M0	2	Malignant	Dpa080082
E3	43	68	F	Pancreas	Duct adenocarcinoma	T2N0M0	2	Malignant	Dpa100492
E4	44	68	F	Pancreas	Duct adenocarcinoma	T2N0M0	2	Malignant	Dpa100492
E5	45	60	M	Pancreas	Duct adenocarcinoma	T3N0M0	1	Malignant	Dpa031213
E6	46	60	M	Pancreas	Duct adenocarcinoma	T3N0M0	1	Malignant	Dpa031213
E7	47	60	M	Pancreas	Duct adenocarcinoma	T3N0M0	3	Malignant	Dpa031219
E8	48	60	M	Pancreas	Duct adenocarcinoma	T3N0M0	3	Malignant	Dpa031219
E9	49	60	M	Pancreas	Duct adenocarcinoma (sparse)	T2N0M0	-	Malignant	Dpa090148
E10	50	60	M	Pancreas	Duct adenocarcinoma	T2N0M0	2–3	Malignant	Dpa090148
F1	51	58	F	Pancreas	Duct adenocarcinoma	T3N1M0	1	Malignant	Dpa090144
F2	52	58	F	Pancreas	Duct adenocarcinoma	T3N1M0	1	Malignant	Dpa090144
F3	53	71	F	Pancreas	Duct adenocarcinoma	T2N0M0	-	Malignant	Dpa090006
F4	54	71	F	Pancreas	Duct adenocarcinoma	T2N0M0	2	Malignant	Dpa090006
F5	55	51	M	Pancreas	Duct adenocarcinoma	T3N1M1	2–3	Malignant	Dpa080277
F6	56	51	M	Pancreas	Duct adenocarcinoma (sparse)	T3N1M1	2	Malignant	Dpa080277
F7	57	47	F	Pancreas	Duct adenocarcinoma	T3N1M0	2–3	Malignant	Dpa031524
F8	58	47	F	Pancreas	Duct adenocarcinoma	T3N1M0	2–3	Malignant	Dpa031524
F9	59	51	F	Pancreas	Duct adenocarcinoma	T3N0M0	2	Malignant	Dpa041842
F10	60	51	F	Pancreas	Duct adenocarcinoma	T3N0M0	2	Malignant	Dpa041842
G1	61	54	M	Pancreas	Duct adenocarcinoma	T2N0M0	2	Malignant	Dpa090151
G2	62	54	M	Pancreas	Duct adenocarcinoma	T2N0M0	2	Malignant	Dpa090151
G3	63	56	M	Pancreas	Duct adenocarcinoma	T2N0M0	3	Malignant	Dpa041019
G4	64	56	M	Pancreas	Duct adenocarcinoma	T2N0M0	3	Malignant	Dpa041019
G5	65	62	M	Pancreas	Duct adenocarcinoma	T3N0M0	-	Malignant	Dpa040685
G6	66	62	M	Pancreas	Duct adenocarcinoma	T3N0M0	3	Malignant	Dpa040685
G7	67	53	F	Pancreas	Duct adenocarcinoma with necrosis (sparse)	T2N0M0	3	Malignant	Dpa041349

G8	68	53	F	Pancreas	Duct adenocarcinoma with necrosis (sparse)	T2N0M0	3	Malignant	Dpa041349
G9	69	78	M	Pancreas	Duct adenocarcinoma	T2N0M0	3	Malignant	Dpa040361
G10	70	78	M	Pancreas	Duct adenocarcinoma	T2N0M0	3	Malignant	Dpa040361
H1	71	48	F	Pancreas	Duct adenocarcinoma	T3N0M0	3	Malignant	Dpa051007
H2	72	48	F	Pancreas	Duct adenocarcinoma	T3N0M0	3	Malignant	Dpa051007
H3	73	66	F	Pancreas	Duct adenocarcinoma	T2N1M0	3	Malignant	Dpa090174
H4	74	66	F	Pancreas	Duct adenocarcinoma	T2N1M0	3	Malignant	Dpa090174
H5	75	55	F	Pancreas	Duct adenocarcinoma	T2N0M0	3	Malignant	Dpa090171
H6	76	55	F	Pancreas	Duct adenocarcinoma	T2N0M0	3	Malignant	Dpa090171
I1	81	48	F	Pancreas	Adjacent normal pancreas tissue	-	-	NAT	Dpa050452
I2	82	48	F	Pancreas	Adjacent normal pancreas tissue	-	-	NAT	Dpa050452
I3	83	31	M	Pancreas	Adjacent normal pancreas tissue	-	-	NAT	Dpa050632
I4	84	31	M	Pancreas	Adjacent normal pancreas tissue	-	-	NAT	Dpa050632
I5	85	51	F	Pancreas	Adjacent normal pancreas tissue	-	-	NAT	Dpa051559
I6	86	51	F	Pancreas	Adjacent normal pancreas tissue	-	-	NAT	Dpa051559
I7	87	51	M	Pancreas	Adjacent normal pancreas tissue	-	-	NAT	Dpa050664
I8	88	51	M	Pancreas	Adjacent normal pancreas tissue	-	-	NAT	Dpa050664
I9	89	52	M	Pancreas	Adjacent normal pancreas tissue (duct adenocarcinoma)	-	2	NAT	Dpa050998
I10	90	52	M	Pancreas	Adjacent normal pancreas tissue	-	-	NAT	Dpa050998
J1	91	47	M	Pancreas	Pancreas tissue	-	-	Normal	Dpa06N004
J2	92	47	M	Pancreas	Pancreas tissue	-	-	Normal	Dpa06N004
J3	93	50	M	Pancreas	Pancreas tissue	-	-	Normal	Dpa06N011
J4	94	50	M	Pancreas	Pancreas tissue	-	-	Normal	Dpa06N011
J5	95	21	F	Pancreas	Pancreas tissue	-	-	Normal	Dpa05N001
J6	96	21	F	Pancreas	Pancreas tissue	-	-	Normal	Dpa05N001
J7	97	27	M	Pancreas	Pancreas tissue	-	-	Normal	Dpa08N047
J8	98	27	M	Pancreas	Pancreas tissue	-	-	Normal	Dpa08N047
J9	99	40	M	Pancreas	Pancreas tissue	-	-	Normal	Dpa05N005
J10	100	40	M	Pancreas	Pancreas tissue	-	-	Normal	Dpa05N005

Table S3. Characteristics of the cohort used for assessing the expression of APLP2.

Position	No.	Age	Sex	Organ/Anatomic Site	Pathology Diagnosis	TNM	Grade	Stage	Type
A1	1	63	F	Pancreas	Ductal adenocarcinoma	T1N0M0	II	1A	Malignant
A2	2	63	F	Perilesional tissue	NAT				NAT
A3	3	51	M	Pancreas	Ductal adenocarcinoma	T1N0M0	II	1A	Malignant
A4	4	51	M	Perilesional tissue	NAT				NAT
A5	5	65	M	Pancreas	Ductal adenocarcinoma	T1N0M0	II	1A	Malignant
A6	6	65	M	Perilesional tissue	NAT				NAT
A7	7	84	M	Pancreas	Ductal adenocarcinoma	T1N0M0	I–III	1A	Malignant
A8	8	84	M	Perilesional tissue	NAT				NAT
A9	9	70	M	Pancreas	Ductal adenocarcinoma	T1N0M0	II	1A	Malignant
A10	10	70	M	Perilesional tissue	NAT				NAT
A11	11	49	M	Pancreas	Ductal adenocarcinoma	T2N0M0	II–III	1B	Malignant
A12	12	49	M	Perilesional tissue	NAT				NAT
A13	13	52	M	Pancreas	Ductal adenocarcinoma	T2N0M0	II	1B	Malignant
A14	14	52	M	Perilesional tissue	NAT				NAT
A15	15	70	F	Pancreas	Ductal adenocarcinoma	T2N0M0	II	1B	Malignant
A16	16	70	F	Perilesional tissue	NAT				NAT
B1	17	54	M	Pancreas	Ductal adenocarcinoma	T2N0M0	II	1B	Malignant
B2	18	54	M	Perilesional tissue	NAT				NAT
B3	19	63	M	Pancreas	Ductal adenocarcinoma	T2N0M0	II	1B	Malignant
B4	20	63	M	Perilesional tissue	NAT				NAT
B5	21	65	M	Pancreas	Ductal adenocarcinoma	T2N0M0	II	1B	Malignant
B6	22	65	M	Perilesional tissue	NAT				NAT
B11	27	71	M	Pancreas	Ductal adenocarcinoma	T2N0M0	II	1B	Malignant
B12	28	71	M	Perilesional tissue	NAT				NAT
B13	29	56	F	Pancreas	Ductal adenocarcinoma	T2N0M0	II	1B	Malignant
B14	30	56	F	Perilesional tissue	NAT				NAT
B15	31	52	M	Pancreas	Ductal adenocarcinoma	T2N0M0	II	1B	Malignant
B16	32	52	M	Perilesional tissue	NAT				NAT
C1	33	57	M	Pancreas	Ductal adenocarcinoma	T2N0M0	II	1B	Malignant
C2	34	57	M	Perilesional tissue	NAT				NAT
C3	35	51	M	Pancreas	Ductal adenocarcinoma	T2N0M0	II	1B	Malignant
C4	36	51	M	Perilesional tissue	NAT				NAT
C5	37	62	F	Pancreas	Ductal adenocarcinoma	T2N0M0	II	1B	Malignant
C6	38	62	F	Perilesional tissue	NAT				NAT
C7	39	55	F	Pancreas	Ductal adenocarcinoma	T2N0M0	II	1B	Malignant
C8	40	55	F	Perilesional tissue	NAT				NAT
C9	41	46	F	Pancreas	Ductal adenocarcinoma	T2N0M0	II	1B	Malignant
C10	42	46	F	Perilesional tissue	NAT				NAT

C11	43	69	M	Pancreas	Ductal adenocarcinoma	T2N0M0	II–III	1B	Malignant
C12	44	69	M	Perilesional tissue	NAT				NAT
C13	45	44	M	Pancreas	Ductal adenocarcinoma	T2N0M0	I–II	1B	Malignant
C14	46	44	M	Perilesional tissue	NAT				NAT
C15	47	64	M	Pancreas	Ductal adenocarcinoma	T2N0M0	II	1B	Malignant
C16	48	64	M	Perilesional tissue	NAT				NAT
D1	49	46	M	Pancreas	Ductal adenocarcinoma	T2N0M0	I–II	1B	Malignant
D2	50	46	M	Perilesional tissue	NAT				NAT
D3	51	52	F	Pancreas	Ductal adenocarcinoma	T2N0M0	II	1B	Malignant
D4	52	52	F	Perilesional tissue	NAT				NAT
D5	53	72	M	Pancreas	Ductal adenocarcinoma	T2N0M0	II–III	1B	Malignant
D6	54	72	M	Perilesional tissue	NAT				NAT
D7	55	71	M	Pancreas	Ductal adenocarcinoma	T2N0M0	II	1B	Malignant
D8	56	71	M	Perilesional tissue	NAT				NAT
D9	57	64	F	Pancreas	Ductal adenocarcinoma	T2N0M0	I–II	1B	Malignant
D10	58	64	F	Perilesional tissue	NAT				NAT
D13	61	77	F	Pancreas	Ductal adenocarcinoma	T2N0M0	II–III	1B	Malignant
D14	62	77	F	Perilesional tissue	NAT				NAT
D15	63	61	M	Pancreas	Ductal adenocarcinoma	T2N0M0	II–III	1B	Malignant
D16	64	61	M	Perilesional tissue	NAT				NAT
E1	65	62	F	Pancreas	Ductal adenocarcinoma	T2N0M0	II	1B	Malignant
E2	66	62	F	Perilesional tissue	NAT				NAT
E5	69	48	M	Pancreas	Ductal adenocarcinoma	T2N0M0	II	1B	Malignant
E6	70	48	M	Perilesional tissue	NAT				NAT
E7	71	64	M	Pancreas	Ductal adenocarcinoma	T2N0M0	II	1B	Malignant
E8	72	64	M	Perilesional tissue	NAT				NAT
E9	73	52	M	Pancreas	Ductal adenocarcinoma	T2N0M0	II–III	1B	Malignant
E10	74	52	M	Perilesional tissue	NAT				NAT
E15	79	63	F	Pancreas	Ductal adenocarcinoma	T2N0M0	II	1B	Malignant
E16	80	63	F	Perilesional tissue	NAT				NAT
F1	81	55	F	Pancreas	Ductal adenocarcinoma	T2M0	II	1/2/2016	Malignant
F2	82	55	F	Perilesional tissue	NAT				NAT
F3	83	62	F	Pancreas	Ductal adenocarcinoma	T2M0	II–III	1–2	Malignant
F4	84	62	F	Perilesional tissue	NAT				NAT
F5	85	78	F	Pancreas	Ductal adenocarcinoma	T3N0M0	II	2A	Malignant
F6	86	78	F	Perilesional tissue	NAT				NAT
F7	87	63	M	Pancreas	Ductal adenocarcinoma	T3N0M0	II	2A	Malignant
F8	88	63	M	Perilesional tissue	NAT				NAT
F11	91	55	M	Pancreas	Ductal adenocarcinoma	T3N0M0	II	2A	Malignant
F12	92	55	M	Perilesional tissue	NAT				NAT
F13	93	60	M	Pancreas	Ductal adenocarcinoma	T3N0M0	II–III	2A	Malignant

F14	94	60	M	Perilesional tissue	NAT				NAT
F15	95	64	M	Pancreas	Ductal adenocarcinoma	T1N1M0	II	2B	Malignant
F16	96	64	M	Perilesional tissue	NAT				NAT
G1	97	52	F	Pancreas	Ductal adenocarcinoma	T1N1M0	II	2B	Malignant
G2	98	52	F	Perilesional tissue	NAT				NAT
G3	99	69	M	Pancreas	Ductal adenocarcinoma	T2N1M0	II	2B	Malignant
G4	100	69	M	Perilesional tissue	NAT				NAT
G5	101	61	M	Pancreas	Ductal adenocarcinoma	T2N1M0	III	2B	Malignant
G6	102	61	M	Perilesional tissue	NAT				NAT
G7	103	52	M	Pancreas	Ductal adenocarcinoma	T2N1M0	II	2B	Malignant
G8	104	52	M	Perilesional tissue	NAT				NAT
G9	105	57	F	Pancreas	Ductal adenocarcinoma	T2N1M0	II	2B	Malignant
G10	106	57	F	Perilesional tissue	NAT				NAT
G11	107	60	F	Pancreas	Ductal adenocarcinoma	T2N1M0	II–III	2B	Malignant
G12	108	60	F	Perilesional tissue	NAT				NAT
G13	109	65	M	Pancreas	Ductal adenocarcinoma	T2N1M0	III	2B	Malignant
G14	110	65	M	Perilesional tissue	NAT				NAT
G15	111	65	M	Pancreas	Ductal adenocarcinoma	T2N1M0	II	2B	Malignant
G16	112	65	M	Perilesional tissue	NAT				NAT
H1	113	41	M	Pancreas	Ductal adenocarcinoma	T2N1M0	II–III	2B	Malignant
H2	114	41	M	Perilesional tissue	NAT				NAT
H3	115	64	M	Pancreas	Ductal adenocarcinoma	T2N1M0	II	2B	Malignant
H4	116	64	M	Perilesional tissue	NAT				NAT
H5	117	55	F	Pancreas	Ductal adenocarcinoma	T2N1M0	II	2B	Malignant
H6	118	55	F	Perilesional tissue	NAT				NAT
H7	119	71	M	Pancreas	Ductal adenocarcinoma	T2N1M0	II	2B	Malignant
H8	120	71	M	Perilesional tissue	NAT				NAT
H9	121	62	M	Pancreas	Ductal adenocarcinoma	T2N1M0	I–II	2B	Malignant
H10	122	62	M	Perilesional tissue	NAT				NAT
H11	123	73	M	Pancreas	Ductal adenocarcinoma	T2N1M0	II	2B	Malignant
H12	124	73	M	Perilesional tissue	NAT				NAT
H13	125	71	M	Pancreas	Ductal adenocarcinoma	T2N1M0	II	2B	Malignant
H14	126	71	M	Perilesional tissue	NAT				NAT
H15	127	65	M	Pancreas	Ductal adenocarcinoma	T2N1M0	II	2B	Malignant
H16	128	65	M	Perilesional tissue	NAT				NAT
I1	129	44	F	Pancreas	Ductal adenocarcinoma	T2—M0	III	1–2	Malignant
I2	130	44	F	Perilesional tissue	NAT				NAT
I3	131	44	F	Pancreas	Ductal adenocarcinoma	T2N1M0	I–II	2B	Malignant
I4	132	44	F	Perilesional tissue	NAT				NAT
I5	133	62	M	Pancreas	Ductal adenocarcinoma	T2N0M1	II	4	Malignant
I6	134	62	M	Perilesional tissue	NAT				NAT

I7	135	60	F	Pancreas	Ductal adenocarcinoma	T2—M1	III	4	Malignant
I8	136	60	F	Perilesional tissue	NAT				NAT
I11	139	72	M	Pancreas	Ductal adenocarcinoma	T2N1M1	II–III	4	Malignant
I12	140	72	M	Perilesional tissue	NAT				NAT
I13	141	56	F	Pancreas	Ductal adenocarcinoma	T3—M1	II–III	4	Malignant
I14	142	56	F	Perilesional tissue	NAT				NAT
I15	143	60	F	Pancreas	Ductal adenocarcinoma	T3N1M1	II	4	Malignant
I16	144	60	F	Perilesional tissue	NAT				NAT

Table S4. Significantly enriched pathways of 397 upregulated genes from the meta-analysis of five datasets (GSE16515, GSE28735, GSE15471, GSE18670, and GSE41368).

Pathway	Total	Expected	Hits	*p*.Value	FDR
Focal adhesion	200	39	142	3.40E−59	7.38E−57
Pathways in cancer	310	60.5	178	1.93E−53	2.10E−51
Regulation of actin cytoskeleton	182	35.5	115	8.84E−40	6.39E−38
Cell cycle	124	24.2	90	1.47E−38	7.97E−37
HTLV-I infection	199	38.8	111	2.91E−31	1.26E−29
Prostate cancer	87	17	66	2.32E−30	8.41E−29
Chronic myeloid leukemia	73	14.2	57	1.74E−27	5.40E−26
ErbB signaling pathway	87	17	61	5.38E−25	1.46E−23
Pancreatic cancer	69	13.5	52	7.21E−24	1.74E−22
Small cell lung cancer	80	15.6	56	5.75E−23	1.21E−21
Renal cell carcinoma	60	11.7	47	6.15E−23	1.21E−21
Bacterial invasion of epithelial cells	56	10.9	45	7.57E−23	1.37E−21
Endometrial cancer	44	8.58	38	1.19E−21	1.98E−20
Adherens junction	70	13.7	50	3.06E−21	4.75E−20
Progesterone-mediated oocyte maturation	80	15.6	53	4.60E−20	6.65E−19
Acute myeloid leukemia	57	11.1	43	6.04E−20	8.20E−19
Neurotrophin signaling pathway	123	24	69	7.92E−20	1.01E−18
Colorectal cancer	49	9.56	39	1.16E−19	1.40E−18
ECM-receptor interaction	84	16.4	53	1.26E−18	1.43E−17
Glioma	65	12.7	45	2.33E−18	2.52E−17
Insulin signaling pathway	137	26.7	69	1.82E−16	1.88E−15
Gap junction	89	17.4	51	1.88E−15	1.86E−14
TGF-beta signaling pathway	84	16.4	49	2.55E−15	2.41E−14
Shigellosis	47	9.17	34	4.48E−15	4.05E−14
Oocyte meiosis	108	21.1	57	5.95E−15	5.16E−14
Non-small cell lung cancer	52	10.1	36	6.42E−15	5.29E−14
Epstein-Barr virus infection	91	17.8	51	6.59E−15	5.29E−14
Hypertrophic cardiomyopathy (HCM)	25	4.88	23	7.56E−15	5.86E−14

Tight junction	118	23	59	4.73E−14	3.54E−13
Chagas disease (American trypanosomiasis)	89	17.4	49	5.85E−14	4.23E−13
T cell receptor signaling pathway	98	19.1	52	7.26E−14	5.08E−13
Wnt signaling pathway	144	28.1	67	7.67E−14	5.20E−13
Melanoma	68	13.3	41	1.08E−13	7.10E−13
Hepatitis C	100	19.5	52	2.12E−13	1.36E−12
Salmonella infection	72	14	42	2.67E−13	1.65E−12
Fc gamma R-mediated phagocytosis	97	18.9	50	9.74E−13	5.87E−12
Measles	102	19.9	51	2.65E−12	1.51E−11
Toxoplasmosis	93	18.1	48	2.65E−12	1.51E−11
Axon guidance	118	23	55	1.33E−11	7.38E−11
GnRH signaling pathway	94	18.3	47	1.96E−11	1.06E−10
Apoptosis	83	16.2	43	3.21E−11	1.70E−10
Dilated cardiomyopathy	78	15.2	41	5.11E−11	2.64E−10
Chemokine signaling pathway	189	36.9	74	1.36E−10	6.84E−10
Influenza A	107	20.9	49	3.91E−10	1.93E−09
Bladder cancer	29	5.66	21	8.83E−10	4.26E−09
Jak-STAT signaling pathway	99	19.3	45	2.79E−09	1.27E−08
Pathogenic Escherichia coli infection	35	6.83	23	2.79E−09	1.27E−08
Cardiac muscle contraction	12	2.34	12	2.87E−09	1.27E−08
p53 signaling pathway	68	13.3	35	2.87E−09	1.27E−08
Thyroid cancer	28	5.46	20	3.34E−09	1.45E−08
Amoebiasis	46	8.97	27	4.43E−09	1.89E−08
B cell receptor signaling pathway	75	14.6	37	4.62E−09	1.89E−08
Fc epsilon RI signaling pathway	75	14.6	37	4.62E−09	1.89E−08
Leukocyte transendothelial migration	108	21.1	47	7.09E−09	2.85E−08
Long-term potentiation	70	13.7	35	7.75E−09	3.01E−08
MAPK signaling pathway	265	51.7	90	7.76E−09	3.01E−08
Melanogenesis	101	19.7	44	2.09E−08	7.94E−08
RIG-I-like receptor signaling pathway	49	9.56	27	2.89E−08	1.08E−07
Epithelial cell signaling in Helicobacter pylori infection	37	7.22	22	8.98E−08	3.30E−07
Osteoclast differentiation	119	23.2	48	9.13E−08	3.30E−07
SNARE interactions in vesicular transport	27	5.27	18	1.13E−07	4.02E−07
Arrhythmogenic right ventricular cardiomyopathy (ARVC)	12	2.34	11	1.47E−07	5.13E−07
Vascular smooth muscle contraction	109	21.3	44	3.09E−07	1.06E−06
mTOR signaling pathway	45	8.78	24	3.92E−07	1.33E−06
Herpes simplex infection	103	20.1	42	4.15E−07	1.38E−06
Viral myocarditis	26	5.07	16	2.87E−06	9.44E−06
VEGF signaling pathway	76	14.8	32	4.45E−06	1.44E−05
Natural killer cell mediated cytotoxicity	138	26.9	49	5.77E−06	1.84E−05
Toll-like receptor signaling pathway	97	18.9	37	1.30E−05	4.08E−05

Long-term depression	70	13.7	29	1.82E−05	5.64E−05
Prion diseases	21	4.1	13	2.34E−05	7.14E−05
Leishmaniasis	51	9.95	23	2.63E−05	7.94E−05
Type II diabetes mellitus	48	9.36	22	2.89E−05	8.59E−05
Alzheimer's disease	49	9.56	22	4.29E−05	1.26E−04
Basal cell carcinoma	47	9.17	21	6.99E−05	2.02E−04
Calcium signaling pathway	177	34.5	55	1.24E−04	3.54E−04
Cholinergic synapse	95	18.5	34	1.30E−04	3.67E−04
NOD-like receptor signaling pathway	49	9.56	21	1.46E−04	4.06E−04
Vibrio cholerae infection	19	3.71	11	2.38E−04	6.53E−04
Adipocytokine signaling pathway	63	12.3	24	4.39E−04	1.19E−03
Hedgehog signaling pathway	56	10.9	22	4.57E−04	1.23E−03
Dorso-ventral axis formation	12	2.34	8	4.77E−04	1.26E−03
Carbohydrate digestion and absorption	18	3.51	10	7.22E−04	1.89E−03
Pertussis	52	10.1	20	1.13E−03	2.91E−03
African trypanosomiasis	25	4.88	12	1.19E−03	3.03E−03
Glycolysis / Gluconeogenesis	65	12.7	23	1.86E−03	4.69E−03
Notch signaling pathway	47	9.17	18	2.08E−03	5.19E−03
Salivary secretion	49	9.56	18	3.55E−03	8.76E−03
Pancreatic secretion	30	5.85	12	7.57E−03	1.85E−02
Retrograde endocannabinoid signaling	60	11.7	20	7.73E−03	1.86E−02
Endocytosis	101	19.7	30	8.55E−03	2.02E−02
Aldosterone−regulated sodium reabsorption	34	6.63	13	8.58E−03	2.02E−02
Malaria	11	2.15	6	1.02E−02	2.38E−02
Tuberculosis	174	33.9	46	1.43E−02	3.30E−02
Gastric acid secretion	56	10.9	18	1.66E−02	3.80E−02
Circadian rhythm-mammal	22	4.29	9	1.70E−02	3.84E−02
Dopaminergic synapse	124	24.2	34	1.91E−02	4.27E−02
Rheumatoid arthritis	19	3.71	8	2.00E−02	4.42E−02

Table S5. Summary of the CHAT analysis.

Hallmarks of Cancer	Number of Papers *	Candidates Involved in the Hallmarks of Cancer
Invasion and metastasis	5912	AGR2, LAMC2, MMP11, TAGLN2, ADAM9, ANXA2, CDH3, MSLN, SERPINB5, CD82, CLDN18, EPHA2, EZR, FXYD3, GPRC5A, ITGA2, MET, MST1R, SLC2A1
Immune destruction	257	AGR2, MMP11, MSLN, CD82, EPHA2, ITGB6
Cellular energetics	112	AGR2, GAPDH, ADAM9, ANXA2, EPHA2, MET, NQO1, SLC2A1
Replicative immortality	19	AGR2, GAPDH, CD82, EPHA2
Evading growth suppressors	415	AGR2, GAPDH, LAMC2, TAGLN2, ADAM9, ANXA2, APLP2, CDH3, MSLN, EPHA2, GPRC5A, ITGA2, NQO1

Genome instability and mutation	1144	AGR2, GAPDH, LAMC2, TAGLN2, ADAM9, ANXA2, APLP2, CDH3, CLDN18, EPHA2, EZR, FXYD3, GPRC5A, ITGA2, ITGB6, MET, MST1R, NQO1, SLC2A1
Inducing angiogenesis	444	AGR2, GAPDH, LAMC2, MMP11, TAGLN2, ADAM9, ANXA2, MSLN, CD82, CLDN18, EPHA2, FXYD3, ITGA2, MET, NQO1, SLC2A1
Resisting cell death	1623	GAPDH, LAMC2, MMP11, TAGLN2, ADAM9, ANXA2, APLP2, CDH3, MSLN, SERPINB5, CD82, EPHA2, FXYD3, GPRC5A, ITGB6, NQO1, SLC2A1
Sustaining proliferative signaling	858	AGR2, GAPDH, LAMC2, MMP11, TAGLN2, ADAM9, ANXA2, APLP2, CDH3, MSLN, SERPINB5, CD82, CLDN18, EPHA2, FXYD3, GPRC5A, ITGA2, ITGB6, MET, MST1R, NQO1
Tumor promoting inflammation	443	AGR2, GAPDH, LAMC2, TAGLN2, ADAM9, ANXA2, MSLN, SERPINB5, EPHA2, GPRC5A, ITGA2, ITGB6, MST1R, NQO1

* The approximate estimation of the number of papers reported in each hallmark in pancreatic cancer. Search query: “Pancreatic cancer” OR “Pancreatic adenocarcinoma” OR “PDAC”. Search date: April 2018

© 2019 by the authors. Licensee MDPI, Basel, Switzerland. This article is an open access article distributed under the terms and conditions of the Creative Commons Attribution (CC BY) license (http://creativecommons.org/licenses/by/4.0/).
